# EGL-9 Controls *C. elegans* Host Defense Specificity through Prolyl Hydroxylation-Dependent and -Independent HIF-1 Pathways

**DOI:** 10.1371/journal.ppat.1002798

**Published:** 2012-07-05

**Authors:** Lyly G. Luhachack, Orane Visvikis, Amanda C. Wollenberg, Adam Lacy-Hulbert, Lynda M. Stuart, Javier E. Irazoqui

**Affiliations:** Program of Developmental Immunology, Department of Pediatrics, Massachusetts General Hospital, and Department of Pediatrics, Harvard Medical School, Boston, Massachusetts, United States of America; Stanford University, United States of America

## Abstract

Understanding host defense against microbes is key to developing new and more effective therapies for infection and inflammatory disease. However, how animals integrate multiple environmental signals and discriminate between different pathogens to mount specific and tailored responses remains poorly understood. Using the genetically tractable model host *Caenorhabditis elegans* and pathogenic bacterium *Staphylococcus aureus*, we describe an important role for hypoxia-inducible factor (HIF) in defining the specificity of the host response in the intestine. We demonstrate that loss of *egl-9*, a negative regulator of HIF, confers HIF-dependent enhanced susceptibility to *S. aureus* while increasing resistance to *Pseudomonas aeruginosa*. In our attempt to understand how HIF could have these apparently dichotomous roles in host defense, we find that distinct pathways separately regulate two opposing functions of HIF: the canonical pathway is important for blocking expression of a set of HIF-induced defense genes, whereas a less well understood noncanonical pathway appears to be important for allowing the expression of another distinct set of HIF-repressed defense genes. Thus, HIF can function either as a gene-specific inducer or repressor of host defense, providing a molecular mechanism by which HIF can have apparently opposing roles in defense and inflammation. Together, our observations show that HIF can set the balance between alternative pathogen-specific host responses, potentially acting as an evolutionarily conserved specificity switch in the host innate immune response.

## Author Summary

Understanding how animals detect infection and mount appropriate responses is key to treating infection and inflammatory disease. We use the tractable model *Caenorhabditis elegans* to study mechanisms of host defense against pathogenic bacteria. Here we show that hypoxia-inducible factor (HIF) is important for ensuring that the intestinal host response to infection has the appropriate specificity. HIF acts as an inducer and a repressor of host genes in the intestine, and regulation of these opposing activities is genetically separable. One well-understood regulatory pathway requires EGL-9 and VHL-1, negative regulators of HIF, to prevent constitutive expression of host defense genes. Noncanonical pathways are less understood; a recently identified noncanonical pathway requires EGL-9 and SWAN-1. This pathway appears to be more important for lifting the repression of defense genes by HIF-1. Mutants defective in EGL-9 are more susceptible to *S. aureus* but more resistant to the distinct pathogen *P. aeruginosa*, indicating that the defense role of HIF-1 is pathogen-specific. These studies are relevant to mammalian defense because mutations in *hif-1*, *egl-9*, and *vhl-1* homologs in mice have similar effects on intestinal inflammation as in worms, and provide a framework to further explore the role of noncanonical HIF signaling in human infection and inflammatory disease.

## Introduction

In mammalian host defense against infection, discrimination of distinct microbes is thought to occur primarily by differential ligation of Pattern Recognition Receptors (PRRs), such as Toll-like Receptor (TLR) heterodimers, followed by activation of downstream kinase cascades that control NF-κB and AP1 family transcription factors [1]. These transcription factors control the expression of pathogen-specific transcriptional programs of host defense genes [2].

TLR pathways are highly conserved, indicating that they arose early during evolution [3]. Despite this, they are not required for host defense in all multicellular animals, suggesting that other undefined modes of host defense exist [4]. This is the case of nematodes such as *Caenorhabditis elegans*, in which the sole TLR, TOL-1, plays a limited role in host defense against infection [5]–[7], and elements of known TLR pathways, including MyD88 and NF-κB itself, are absent [4], [8]. However, *C. elegans* detects bacterial infection and elicits pathogen-specific host defense responses [5], [9]. A few signaling pathways necessary for *C. elegans* host defense have been partially elucidated, including extracellular signal regulated kinase (ERK) [10], p38 mitogen-activated protein kinase (MAPK) [11], transforming-growth factor β (TGF-β) [6], and β-catenin [12] pathways, but their molecular mechanisms of signal transduction and interactions with other cellular pathways remain largely unknown. Most importantly, absent clear mechanisms for bacterial detection, how *C. elegans* discriminates between distinct pathogens to produce pathogen-tailored responses is not understood.

Hypoxia-inducible factor (HIF) is a highly conserved heterodimeric transcription factor, which is composed of α and β subunits (HIF-1 and AHA-1 in *C. elegans*, respectively) and is best known to mediate cellular responses to low oxygen concentrations, by activating hundreds of genes involved in metabolism, cell division, angiogenesis, iron homeostasis, and apoptosis [13], [14]. As in all species tested so far, an important mechanism of control of *C. elegans* HIF activity is by rapid turnover of the HIF-1 subunit, modulated by canonical hypoxia signaling [13]. Under normal O_2_ levels, HIF-1 is hydroxylated by the prolyl hydroxylase (PHD) EGL-9, which converts HIF-1 to a ligand for von Hippel Lindau protein (VHL-1). VHL-1 binding leads to HIF-1 ubiquitination and degradation by the proteasome [15]–[17]. When O_2_ is scarce, PHD activity diminishes and HIF-1 is stabilized, allowing HIF-1 accumulation, nuclear translocation, recruitment to target promoters by AHA-1, and target gene expression. Additionally, EGL-9 represses HIF by a noncanonical pathway that is independent of EGL-9 PHD activity and of VHL-1, but that requires the protein scaffold SWAN-1 [18], [19]. Therefore, EGL-9 has at least two divergent functions that converge on HIF.

Recently, HIF was also implicated in host defense in mammals and nematodes. Human HIF is activated during infection by bacteria and viruses, and during chronic inflammation [20]. Furthermore, murine and human HIF regulate the expression of inflammatory genes. For instance, HIF is essential in phagocytes for the induction of antimicrobial responses and for prevention of systemic infection, suggesting that HIF has pro-inflammatory roles [21]. Similarly, *C. elegans* HIF promotes host defense against *Pseudomonas aeruginosa* [18], [22], a Gram-negative pathogen of great clinical importance, and against pore-forming toxins from *Bacillus thuringiensis* and *Vibrio cholerae* [22], implying that HIF drives the expression of host defense genes that enhance host survival of infection.

In contrast, HIF deletion increases inflammation in mouse models of intestinal inflammation and of infection by *C. difficile*, suggesting that HIF may also have anti-inflammatory roles [23], [24]. The molecular basis for these opposing effects of HIF on host defense and inflammation is not well understood.

Similar to *P. aeruginosa*, the Gram-positive pathogen *Staphylococcus aureus* infects the intestine and kills *C. elegans* [25]. Furthermore, similar to *B. thuringiensis* and *V. cholerae*, *S. aureus* is known to deploy pore-forming toxins as virulence factors [26]. For these reasons, we hypothesized that hypoxia signaling might play a role in defense against *S. aureus*. Unexpectedly, we found that deletion of *egl-9* conferred enhanced susceptibility to *S. aureus*-mediated killing, which was dependent on *hif-1*. Mechanistically, this effect appeared to be the result of HIF-1-mediated defense gene repression, suggesting that HIF can function both as an inducer and a repressor of distinct sets of host defense genes. Furthermore, canonical hypoxia signaling appeared to be specialized for controlling gene activation by HIF, whereas noncanonical signaling appeared specifically to control gene repression by HIF. These observations provide a rationale for the existence of two parallel HIF-controlling pathways divergent from EGL-9, and identify HIF as an important factor that can modulate the balance between pathogen-specific host responses.

## Results

### *egl-9*/PHD is required for host defense against *S. aureus*

**Figure 1:**
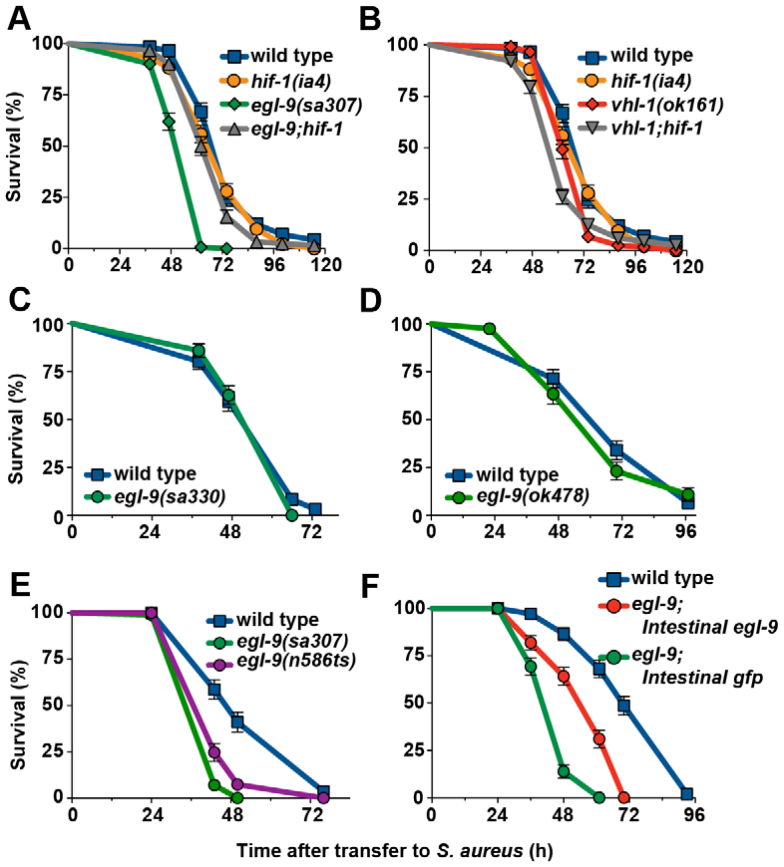
*egl-9* inactivation causes enhanced susceptibility to *S. aureus-*mediated killing. **A.** *egl-9(sa307)* animals exhibited enhanced susceptibility, whereas *egl-9(sa307);hif-1(ia4)* mutants exhibited near wild-type susceptibility. Survival analysis: *egl-9* Kaplan-Meier Median Survival (MS) = 62 h, Time to 50% Death by nonlinear regression analysis (LT_50_) = 48.78 h, Number of animals (N) = 142, *p*<0.0001 (Log-Rank test, compared with wild type); *egl-9;hif-1* MS = 68 h, LT_50_ = 62.10 h, N = 122/2, *p* = 0.0030 (compared with wild type). **B.** *vhl-1(ok161)* and *hif-1(ia4)* animals exhibited near wild-type susceptibility. Survival analysis: wild type MS = 74 h, LT_50_ = 67.03 h, N = 117/5; *vhl-1* MS = 62 h, LT_50_ = 61.86 h, N = 118, *p*<0.0001 (compared with wild type); *hif-1* MS = 74 h, LT_50_ = 64.77 h, N = 136, *p* = 0.0943 (compared with wild type). **C.** *egl-9(sa330)* animals and **D.** *egl-9(ok478)* animals exhibit wild type susceptibility. **E.** *egl-9(n586ts)* animals are hypersusceptible to *S. aureus*. Survival analysis: *egl-9(sa307)* MS = 43 h, N = 95/1, *p*<0.0001 (compared with wild type); *egl-9;(n586ts)* MS = 43 h, N = 96/15, *p*<0.0001 (compared with wild type); wild type MS = 50 h, N = 92/9. As all killing assays, this assay was performed at 25°C, which is the restrictive temperature of *n586ts*. **F.** Wild type, *egl-9(sa307);crp-1::egl-9* (Intestinal *egl-9*), and *egl-9(sa307);crp-1::gfp* (Intestinal *gfp*) animals show that intestinal expression of EGL-9, but not GFP, rescues the *egl-9(sa307)* enhanced susceptibility phenotype. Survival analysis: wild type MS = 70 h, N = 108/7; *Intestinal egl-9* MS = 61 h, N = 115/14, *p*<0.0001 (compared with wild type), *p*<0.0001 (compared with *Intestinal gfp*); *Intestinal gfp* MS = 48 h, N = 102/3, *p*<0.0001 (compared with wild type). Results are representative of two independent trials, performed in triplicate. Animals were subjected to *cdc-25* RNAi to prevent reproduction, and subsequently transferred to *S. aureus* killing assay plates.

To evaluate whether HIF-1 is involved in host defense against *S. aureus*, we infected wild type *C. elegans* and mutants defective in hypoxia signaling, and followed survival over time. Deletion of *egl-9* in *egl-9(sa307)* mutants causes HIF-1 accumulation and constitutive activation [17]. Surprisingly, *egl-9(sa307)* mutants exhibited enhanced susceptibility to *S. aureus-*mediated killing, compared with wild type (**Figure 1A**). Simultaneous deletion of *hif-1* suppressed this effect (**Figure 1A**). In contrast, inactivation of *hif-1* did not substantially alter susceptibility (**Figure 1A**), indicating that although hyperactivation of HIF-1 by deletion of *egl-9* is deleterious for *C. elegans* defense against *S. aureus*, loss of HIF-1 is not sufficient to confer resistance. The susceptibility of *egl-9(sa307)* animals is likely not due to non-specific lack of viability, since uninfected *egl-9(sa307)* mutants are long lived [18], [27], [28] and resistant to a number of abiotic stresses [22].

One mechanism by which EGL-9 represses HIF-1 requires VHL-1, so we examined the susceptibility of *vhl-1(ok161)* mutants to *S. aureus*. Inactivation of *vhl-1* did not alter susceptibility (**Figure 1B**), indicating that VHL-1-independent EGL-9 regulation of HIF-1 (a “noncanonical” pathway) is required for host defense.

**Figure 2:**
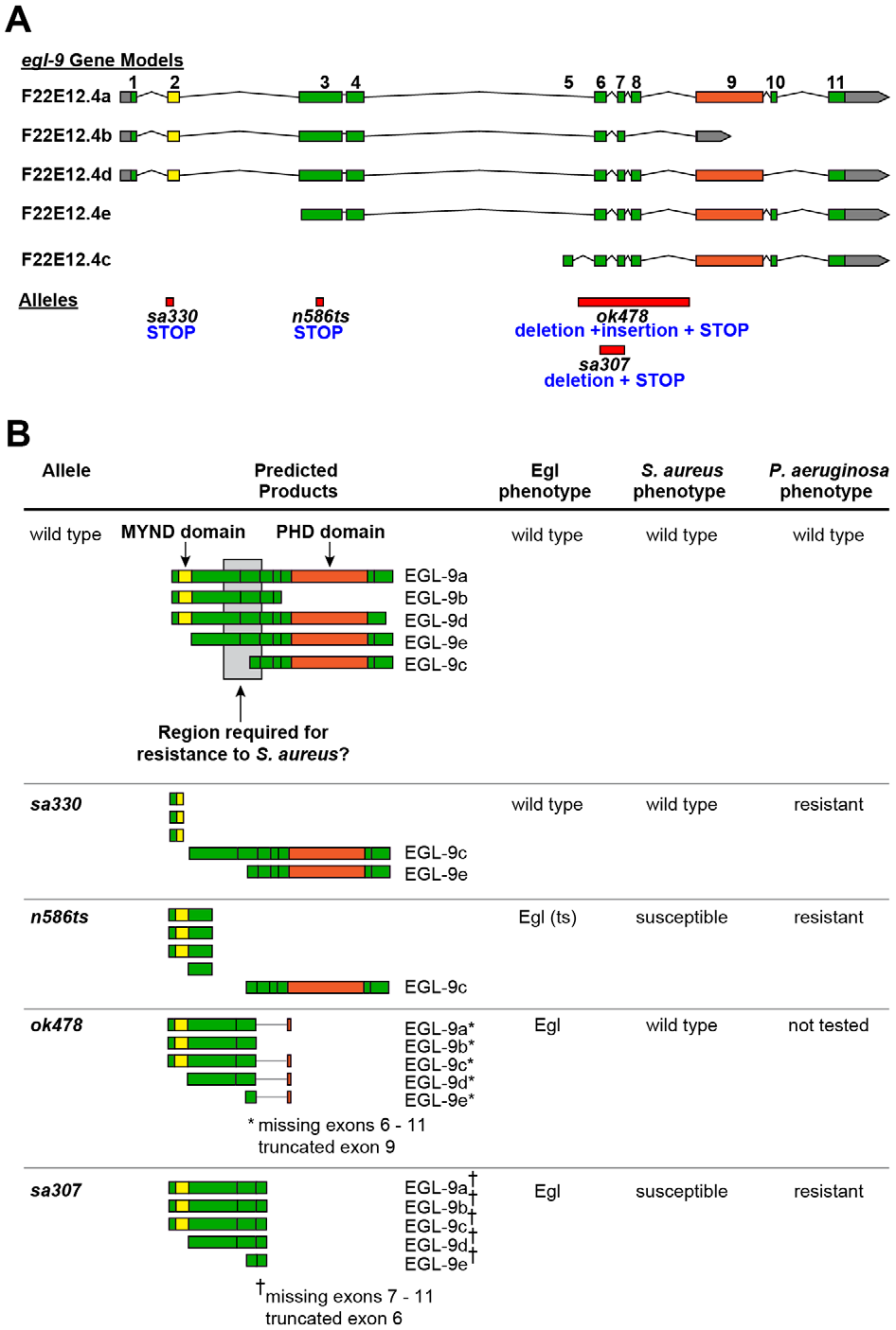
*egl-9* allelic series informs on molecular biology of EGL-9. **A.** *egl-9* gene models extracted from the *C. elegans* genome database (WormBase, www.wormbase.org), indicating exon number and location of mutations. **B.** Predicted protein products from each splice isoform, indicating conserved domains and region determined to be required for wild type susceptibility to *S. aureus*, and summary of phenotypes for each allele, describing predicted protein products in each case. *S. aureus* phenotype as presented in the main text. *P. aeruginosa* phenotype refers to cyanide-mediated killing by *P. aeruginosa* strain PAO1 [18], [32].

To confirm our unexpected findings, we evaluated additional alleles of *egl-9*, which differ in their predicted protein products. The *egl-9* gene comprises eleven exons that are alternatively spliced to produce five different proteins that differ in their domain composition (EGL-9a–e, **Figure 2A,B**) [19]. *egl-9(sa330)* and *egl-9(ok478)* mutants respectively produce either full-length EGL-9c and EGL-9e (containing the C-terminal prolyl hydroxylase, or PHD, domain but not the N-terminal myeloid translocation protein 8, Nervy, and DEAF-1, or MYND, domain) or only truncated fragments (containing the MYND domain but not the PHD domain, **Figure 2B**). These mutants exhibited wild type susceptibility to *S. aureus* (**Figure 1C,D**), indicating that the MYND and PHD domains are dispensable for host defense. Additionally, *egl-9(ok478)* animals exhibit an egg-laying (Egl) defect [19], but not enhanced susceptibility to *S. aureus*, showing that the enhanced susceptibility and Egl phenotypes are separable.

In contrast, *egl-9(n586ts)* mutants exhibited enhanced susceptibility to *S. aureus* (**Figure 1E**). These animals are predicted to produce full-length EGL-9c, which contains only the PHD domain, and truncated fragments that contain only the MYND domain (**Figure 2B**). This observation supports the notion that the MYND and PHD domains are not sufficient for host defense. Collectively, these results suggest that exons 3 and 4 of *egl-9*, which contain a serine-rich region, are important determinants of susceptibility to *S. aureus* (**Figure 2B**). By this model, *sa307*, which generates a premature stop codon just upstream of the PHD domain (**Figure 2A,B**), would not be expected to exhibit enhanced susceptibility; although our results appear to contradict the proposed model, it is possible that the translation of *egl-9(sa307)* transcripts results in misfolded or otherwise inhibited EGL-9 fragments. For subsequent studies we continued to use *egl-9(sa307)*, since it is the most widely used allele.

*S. aureus* infects *C. elegans* through the intestine, where it causes intestinal epithelial cell pathology and lysis, before causing internal organ lysis and nematode death [5]. Intestinal epithelial cells represent the major site of host defense in this system [5]. We therefore tested whether exclusive intestinal epithelial cell expression of *egl-9* could rescue the susceptibility phenotype of *egl-9(sa307)* mutants. While epidermal, muscle, or neuronal expression of *egl-9* had no effect (**Figure S1**), intestinal expression of *egl-9* partially rescued the susceptibility phenotype of *egl-9(sa307)* (**Figure 1F**). Taken together, our results thus far suggested that EGL-9 is required in intestinal epithelial cells to suppress HIF-1-mediated enhanced susceptibility to *S. aureus* by a *vhl-1-*independent, or noncanonical, pathway.

### *hif-1*/HIF-1α is dispensable for host defense gene induction

How does HIF-1 mediate susceptibility to *S. aureus*, and how does EGL-9 suppress that susceptibility? Infection by *S. aureus* triggers a pathogen-specific transcriptional response in *C. elegans*, which enhances host survival [5]. As mentioned, HIF is a heterodimeric transcription factor, composed in *C. elegans* of HIF-1 (HIFα) and AHA-1 (HIFβ). Because loss of *egl-9* caused enhanced susceptibility to *S. aureus* in a HIF-1-dependent manner, we hypothesized that HIF-1 may regulate the transcriptional host response to infection.

To determine the role of HIF-1 in defense gene regulation, we performed qRT-PCR to measure expression of 17 *S. aureus-*induced genes in infected and uninfected wild type and *hif-1* animals. These genes include putative antimicrobials and were selected as markers of the wider host response [5]. *C. elegans* is a natural bacterivore, which is reared in the laboratory by feeding on nonpathogenic *Escherichia coli* isolate OP50. For our transcription profiling experiments, *E. coli* OP50-fed uninfected animals represent the basal, or reference, state. Overall, we did not observe major differences in gene expression between wild type and *hif-1* animals, although *oac-31* and *clec-60* were more highly expressed in uninfected and infected *hif-1* animals, respectively, and *lys-5* was less expressed in both infected and uninfected *hif-1* animals (**Figure S2A**).

Next, we evaluated the induction of the selected marker genes during infection, by comparing infected animals to uninfected controls. As previously shown [5], the marker genes showed a range of induction from 2-fold to 1000-fold in wild type animals (**Figure S2B**). Compared with wild type, *hif-1* mutants displayed similar levels of gene induction, except in the cases of *oac-31*, which trended towards lower induction, and *lys-5*, *cyp-34A4*, and *Y65B4BR.1*, which trended towards higher levels of induction (**Figure S2B**). Therefore, *hif-1* is dispensable for the induction of *S. aureus* host response genes, which is consistent with the wild type susceptibility of *hif-1* mutants (**Figure 1A**). The lack of effect of *hif-1* mutation on survival and defense gene expression was not due to *hif-1* gene repression during infection, because *hif-1* was equally expressed in infected and uninfected wild type animals (**Figure S2C**).

### Activated HIF-1 causes both host defense gene overexpression and repression

**Figure 3:**
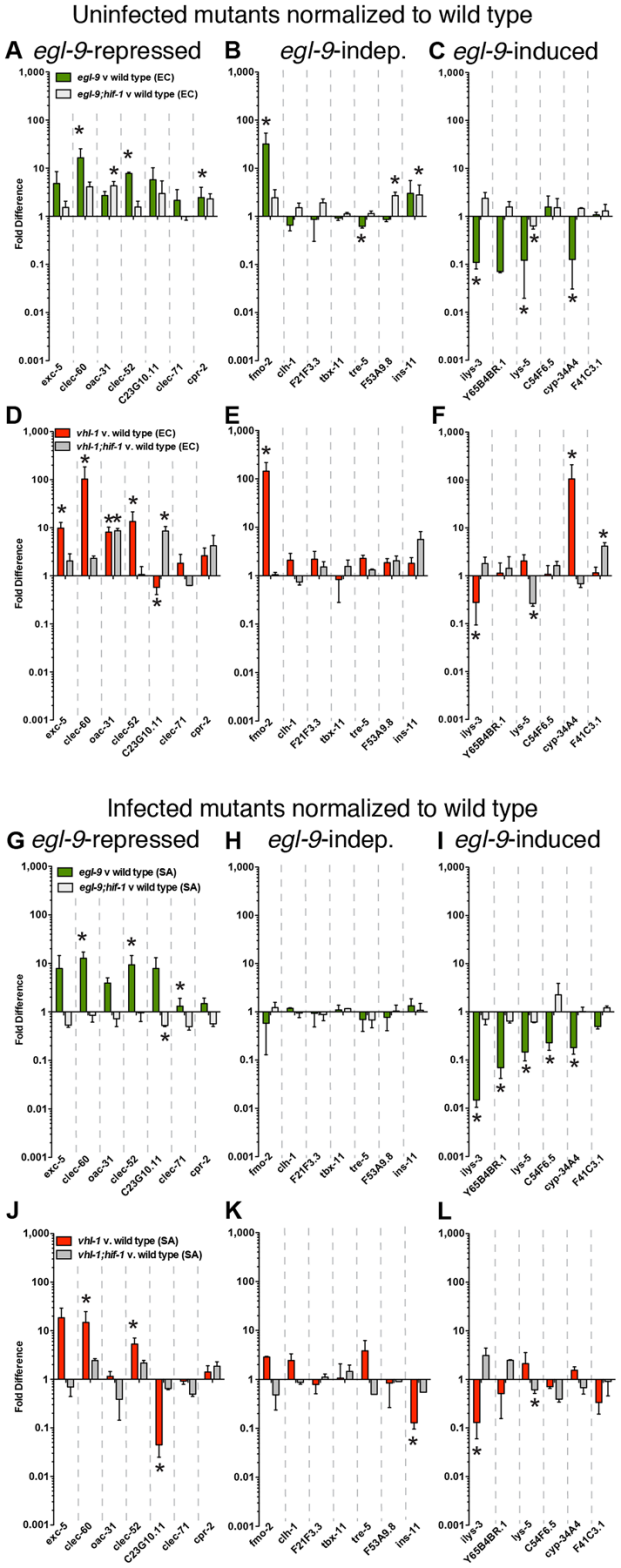
*egl-9* is required to lift repression of host defense genes by *hif-1*. **A, B, C.** *egl-9(sa307)* and *egl-9(sa307);hif-1(ia4)* animals were fed heat-killed non-pathogenic *E. coli* for 8 h and gene expression, measured by qRT-PCR, was normalized to parallel wild type controls. Genes were divided into three groups, according to their expression in infected *egl-9* animals (see G, H, I): **A.** *egl-9-*repressed genes, **B.** *egl-9-*independent genes, and **C.** *egl-9-*induced genes. **D, E, F.** *vhl-1(ok161)* and *vhl-1(ok161);hif-1(ia4)* animals were fed heat-killed non-pathogenic *E. coli* and gene expression was normalized to wild type. Genes were grouped as in A, B, C. **G, H, I.** *egl-9(sa307)* and *egl-9(sa307);hif-1(ia4)* animals were infected with *S. aureus* for 8 h and gene expression, measured by qRT-PCR, was normalized to wild type. Genes are divided into three groups: **G.** *egl-9-*repressed genes, **H.** *egl-9-*independent genes, and **I.** *egl-9-*induced genes. **J, K, L.** *vhl-1(ok161)* and *vhl-1(ok161);hif-1(ia4)* animals were infected and gene expression was normalized to wild type. Genes are grouped as in G, H, I. Data are means of 2–5 independent biological replicates, error bars are SEM. *, *p*≤0.05 (compared with wild type by two-sample *t* test).

As we have shown, lack of *egl-9* confers enhanced susceptibility to *S. aureus* via *hif-1* (**Figure 1A**). We envisioned two possibilities: **a)** hyperactive HIF-1 might cause pathologically high levels of host gene expression during infection, causing enhanced susceptibility due to self-damage, and **b)** hyperactive HIF-1 might repress the host response, causing enhanced susceptibility due to deficient host defense. To discriminate between these scenarios, we measured marker gene expression by qRT-PCR in uninfected (**Figure 3A–C**) and infected (**Figure 3G–L**) *egl-9* and *egl-9;hif-1* double mutants relative to wild type controls. According to their expression during infection, we found three subsets of genes: **1)** genes whose expression was increased in *egl-9* mutants, including C-type lectin genes *clec-60*, *clec-52*, and *clec-71* (“*egl-9-*repressed genes”, **Figure 3A, G**), **2)** genes whose expression did not change, such as flavin-containing mono-oxygenase *fmo-2* and putative antimicrobial peptide *F53A9.8* (“*egl-9-*independent genes”, **Figure 3B, H**), and **3)** genes whose expression was reduced, including antimicrobials such as lysozymes (*ilys-3* and *lys-5*) and secreted phospholipase *Y65B4BR.1* (“*egl-9-*induced genes”, **Figure 3C, I**). Many of these expression changes also occurred in uninfected animals (**Figure 3** **A–C**), indicating that basal expression could also be altered by *egl-9* mutation, independently of infection. The majority of gene expression changes were *hif-1-*dependent (except for *oac-31*, *cpr-2*, *ins-11*, and *lys-5* in uninfected animals), implicating HIF-1 as both activator and repressor of the intestinal host response to infection (**Figure 3A–C, G–I**). Collectively, these results show that *egl-9* inactivation causes *hif-1-*dependent up- and down-regulation of distinct sets of host defense genes in both uninfected and infected states.

EGL-9 blocks HIF-1 by a canonical pathway that involves VHL-1-dependent ubiquitination and subsequent degradation [19]. To test whether inactivation of the canonical pathway is sufficient for the gene expression changes observed in *egl-9* mutants, we measured gene expression in *vhl-1* and *vhl-1;hif-1* animals relative to wild type. Interestingly, mutation of *vhl-1* caused significant upregulation of two out of three *egl-9-*repressed genes (*clec-60* and *clec-52*, with an additional four genes trending higher than wild type in uninfected animals, and two in infected animals). These changes were also HIF-1-dependent (except for *oac-31*, **Figure 3D, J**). Similarly, *egl-9*-independent genes were mostly unchanged in *vhl-1* mutants (except for *fmo-2* in uninfected animals and *ins-11* in infected animals, **Figure 3E, K**). In contrast, most *egl-9-*induced genes remained unchanged in *vhl-1* mutants (except for *ilys-3* and *cyp-34A4*, **Figure 3F, L**). Thus, *vhl-1* inactivation appeared to cause similar gene upregulation as *egl-9*, but not to cause repression of *egl-9*-induced genes. This result implies that VHL-1-mediated canonical signaling is important for preventing HIF-1-induced gene activation, but not HIF-1-mediated gene repression.

Mutations in *egl-9* or *vhl-1* could lead to increased expression of defense genes in intestinal cells, or alternatively could lead to ectopic expression in additional tissues. To better understand the locus of defense gene overexpression, we used animals expressing GFP driven by the *clec-60* promoter [12]. We observed progressively higher expression in the intestinal epithelial cells of wild type, *hif-1*, *egl-9*, and *vhl-1* animals, as predicted by qRT-PCR (**Figure 3A,D** **,** **4** **, S3**). In uninfected *egl-9* and *vhl-1* animals, we also observed very low ectopic expression in the excretory cell, visible after long exposure (**Figure S4**). These results suggest that defense gene overexpression in *egl-9* and *vhl-1* mutants occurs largely in the intestinal epithelium, rather than ectopically in other tissues.

**Figure 4:**
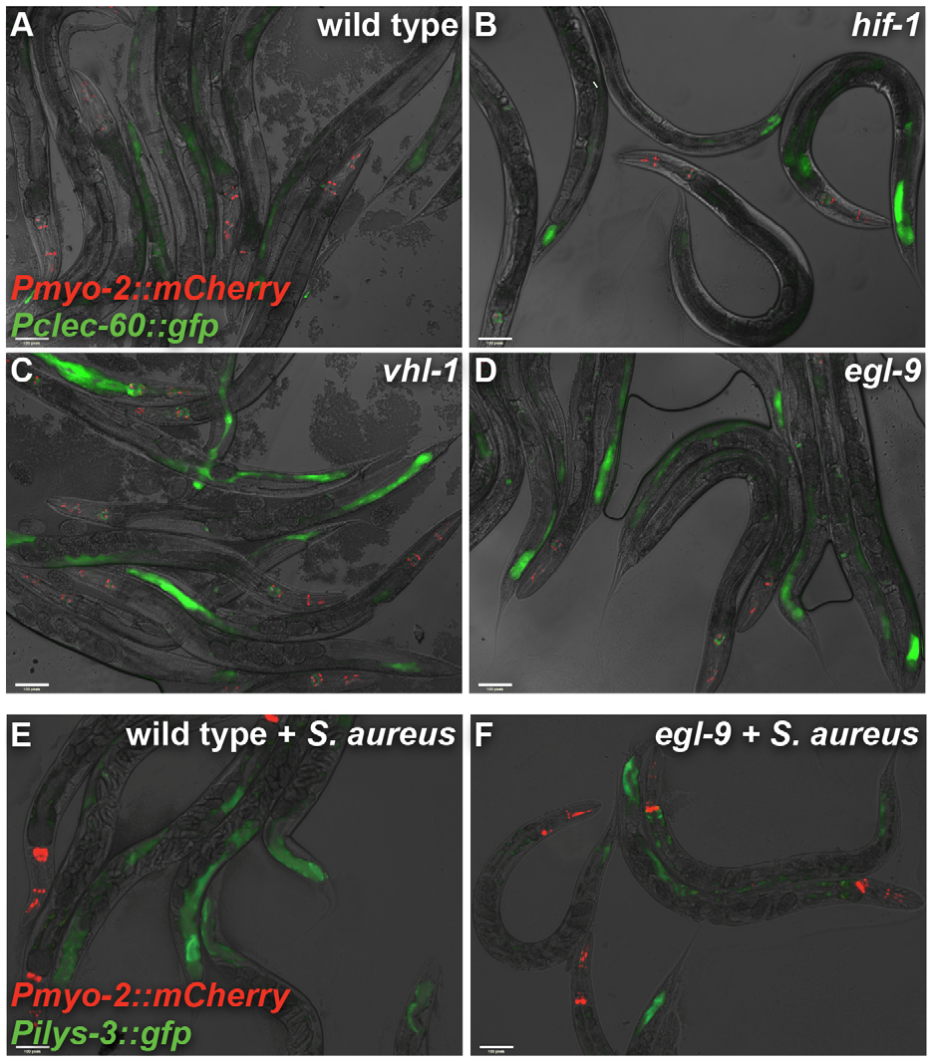
*vhl-1* and *egl-9* affect defense gene expression in the intestinal epithelium. **A, B, C, D.** *clec-60::gfp* expression in uninfected wild type (A), *hif-1(ia4)* (B), *vhl-1(ok161)* (C), and *egl-9(sa307)* (D) animals. Note increased GFP intensity and number of intestinal cells expressing GFP in *vhl-1* and *egl-9* animals. Results are quantified and compared in Figure S3. **E, F.** *ilys-3::gfp* expression in wild type (E) and *egl-9(sa307)* (F) animals infected with *S. aureus* for 24 h. Note decreased intensity and intestinal domain of GFP expression in *egl-9* animals. Red, *Pmyo-2::mCherry* co-injection marker expressed in the pharynx.

To verify the repression of *ilys-3* in *egl-9* mutants, we used animals carrying *ilys-3* promoter-driven GFP. This construct was highly expressed in intestinal epithelial cells of infected wild type animals, but not in infected *egl-9* animals, as predicted by qRT-PCR (**Figure 3I** **,** **4E,F**), confirming that *ilys-3* induction in the intestine requires *egl-9* function.

To further test our conclusion that canonical HIF-1 regulation is important to prevent defense gene overexpression, we evaluated gene expression in transgenic *hif-1* animals overexpressing either wild type HIF-1 or a mutant HIF-1 allele (*hif-1^P621G^*) in which proline 621 is mutated to glycine, abrogating EGL-9-mediated prolyl hydroxylation [17], [19]. In animals expressing *hif-1^P621G^*, canonical EGL-9 regulation of HIF-1 is disrupted (causing HIF-1 accumulation) but noncanonical HIF-1 regulation is presumably functional. Thus, we would expect the expression profile of animals expressing *hif-1^P621G^* to be different from that of animals expressing wild type *hif-1*, and similar to that of *vhl-1* mutants.

**Figure 5:**
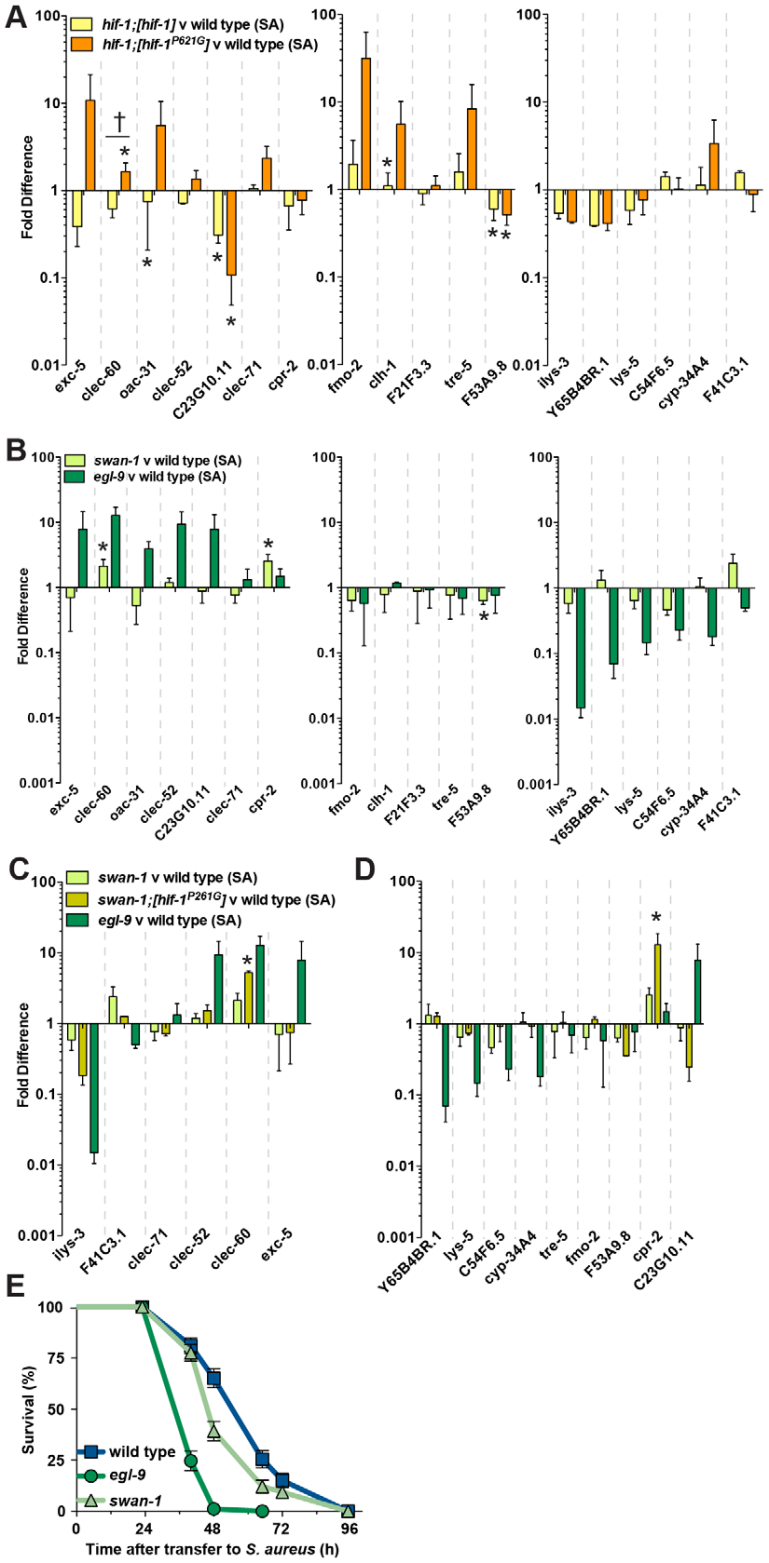
Noncanonical signaling contributes to lifting *hif-1*-mediated repression of the host defense response. **A.** *hif-1* animals overexpressing wild type HIF-1 (*hif-1;[hif-1]*) or non-hydroxylatable HIF-1 (*hif-1;[hif-1^P621G^]*) were infected with *S. aureus* for 8 h and gene expression, measured by qRT-PCR, was normalized to wild type. Data are means of 2 independent biological replicates, error bars are SEM. *, *p*≤0.05 (compared with wild type by two-sample *t* test); †, *p*≤0.05 (compared *hif-1;[hif-1]* with *hif-1;[hif-1^P621G^]* by two-sample *t* test). **B.** *swan-1(ok267)* mutants were infected with *S. aureus* for 8 h and gene expression, measured by qRT-PCR, was normalized to wild type. *egl-9(sa307)* data from Figure 3 are included for comparison. Results are means of 3–5 independent biological replicates, error bars are SEM. *, *p*≤0.05 (compared with wild type by two-sample *t* test). **C.** Genes whose expression levels were intermediate in *swan-1; [hif-1^P621G^]* animals compared with *swan-1* and *egl-9* animals. *swan-1* animals overexpressing non-hydroxylatable HIF-1 (*swan-1;hif-1^P621G^*) were infected with *S. aureus* for 8 h and gene expression, measured by qRT-PCR, was normalized to wild type. Data are means of 2 independent biological replicates, error bars are SEM. *, *p*≤0.05 (compared with wild type by two-sample *t* test). Data for *swan-1* and *egl-9* mutants from Figure 5A and 3 are included for comparison. **D.** Genes whose expression levels did not appear intermediate in *swan-1;hif-1^P621G^* animals compared with *egl-9* and *swan-1* animals. Data for *swan-1* and *egl-9* mutants from Figure 5A and 3 are included for comparison. **E.** *swan-1(ok267)* mutants exhibit enhanced susceptibility to *S. aureus*. Survival analysis: wild type MS = 65 h, N = 110/4; *swan-1* MS = 48 h, N = 108/1, *p* = 0.0036 (compared with wild type); *egl-9* MS = 40 h, N = 87/2, *p*<0.0001 (compared with wild type). Results are representative of two independent trials, performed in triplicate.

Relative to infected wild type animals, overexpression of wild type HIF-1 in infected animals caused significant repression of *oac-31*, *C23G10.11*, and *tre-5*, and an overall trend towards repression of both *egl-9-*repressed and -induced genes (10 out of 18 genes, **Figure 5A**), suggesting that overexpression of HIF-1 is sufficient to cause gene expression changes. On the other hand, overexpression of HIF-1*^P621G^* caused significantly higher expression only of *clec-60* (similar to *vhl-1* mutants). Although the results did not exactly mirror those obtained for *vhl-1* mutants (**Figure 3J–L**), we observed a trend towards increased expression of a subset of genes, including *exc-5*, *clec-52*, *fmo-2*, *clh-1*, *tre-5*, and *cyp-34A4* (**Figure 5A**), as well as significant repression of *C23G10.11*, similar to *vhl-1* mutants (**Figure 3J–L**). These results support the notion that canonical EGL-9 signaling, acting through HIF-1 hydroxylation and VHL-1-mediated degradation, blocks HIF-mediated defense gene activation.

### A noncanonical pathway inhibits HIF-1-mediated defense gene repression

Recently, a noncanonical pathway of HIF-1 inhibition by EGL-9 was described in *C. elegans* [19]. This noncanonical pathway, postulated to act within the nucleus [19], does not require EGL-9 catalytic activity and operates independently of VHL-1. Instead, the described noncanonical HIF inhibition depends on scaffold protein SWAN-1 [18] (known as SWAN-1/DCAF7/HAN11/WDR68 in mammals). *swan-1* mutation synergizes with loss of *vhl-1* and leads to higher HIF-1 activity than in *vhl-1* single mutants. However, *swan-1* mutation alone does not cause HIF-1 accumulation, suggesting a model whereby SWAN-1 influences HIF-1 transcriptional activity [18]. Based on the fact that genes *Y65B4BR.1*, *lys-5*, *C54F6.5*, and *cyp-34A4* were repressed in *egl-9* but not in *vhl-1* mutants (**Figure 3I,L**), we hypothesized that noncanonical HIF-1 control was important for regulating *egl-9-*induced (*hif-1-*repressed) defense genes. To test the hypothesis that SWAN-1, which is involved in noncanonical HIF control, plays a role in lifting HIF-1-mediated gene repression, we evaluated gene expression in infected *swan-1* animals compared with wild type. Loss of *swan-1* caused minor differences in gene expression relative to wild type, with an overall trend towards repression (**Figure 5B**). This result suggested that, although noncanonical signaling is important for lifting host defense gene repression by HIF-1, SWAN-1 plays a limited role in the regulation of the small set of genes we evaluated.

Next, we combined *swan-1* and *hif-1^P621G^* mutations to perturb both known pathways of HIF inhibition and test the extent to which the double mutants phenocopy the gene expression profile of *egl-9* mutants. Infected *swan-1;hif-1^P621G^* double mutants exhibited complex gene expression profiles compared with *swan-1* and *egl-9* single mutants. The expression of a subset of genes was intermediate between *swan-1* and *egl-9* mutants, suggesting a trend towards the *egl-9* profile by impairment of canonical signaling in *swan-1* mutants (**Figure 5C**). However, a second subset of genes were not intermediate (*e.g. lys-5* and *cpr-2*, **Figure 5D**). Similar effects were observed in uninfected animals (**Figure S5**). Thus, combination of *swan-1* and *hif-1^P621G^* mutations was not sufficient to generate an expression profile similar to *egl-9* mutants, suggesting that stabilization of HIF-1 and loss of *swan-1* are insufficient to phenocopy mutation of *egl-9*. Presumably, additional pathway components function downstream of EGL-9 for noncanonical control of HIF-1-mediated repression of defense genes.

Because the observed defense gene repression in *swan-1* mutants was relatively small compared with *egl-9* animals, we sought to independently evaluate the biological relevance of the known noncanonical pathway, dependent on *swan-1*, to host defense against *S. aureus*. We found that mutation of *swan-1* was sufficient to confer enhanced susceptibility (**Figure 5E**). The magnitude of the effect was less than that seen in *egl-9* mutants, which is consistent with the smaller effect of *swan-1* on gene expression (**Figure 5B**). This result suggests that, despite having a minor role in the expression of the small set of genes we tested, *swan-1* has an important role in host defense.

**Figure 6:**
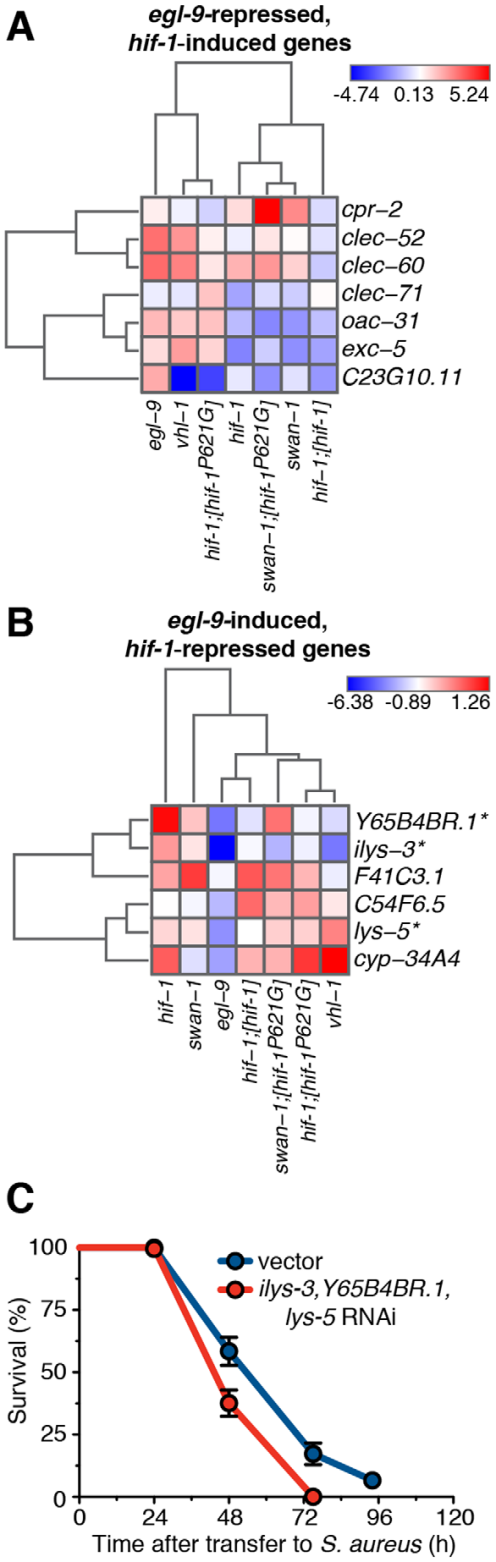
Repression of HIF-1-repressed host defense genes causes enhanced susceptibility to *S. aureus*. **A.** Non-hierarchical cluster analysis of *egl-9-*induced gene expression changes in infected *hif-1(ia4)*, *swan-1(ok267)*, *swan-1(ok267);[hif-1^P621G^] egl-9(sa307)*, *vhl-1(ok161)*, *hif-1(ia4);[hif-1^P621G^]*, and *hif-1;[hif-1]* animals normalized to wild type. **B.** Non-hierarchical cluster analysis of *egl-9-*repressed gene expression changes in infected *hif-1(ia4)*, *swan-1(ok267)*, *swan-1(ok267);[hif-1^P621G^] egl-9(sa307)*, *vhl-1(ok161)*, *hif-1(ia4);[hif-1^P621G^]*, and *hif-1;[hif-1]* animals normalized to wild type. Blue indicates downregulation, red indicates upregulation. Color intensity reflects magnitude of change; darker colors correspond to larger changes. **C.** Enhanced-RNAi mutant *eri-1(mg366)* animals were subjected to feeding RNAi from hatching to L4 stage, and subsequently transferred to *S. aureus* pathogenesis assays. Vector, empty L4440 RNAi plasmid. Survival analysis: *vector* MS = 75 h, N = 83/12; *ilys-3*, *Y65B4BR.1*, *lys-5* RNAi MS = 48 h, N = 93/9, *p* = 0.0001 (compared with vector control). Results are representative of two independent trials, performed in triplicate.

To better detect large-scale trends across our different experiments, we performed non-hierarchical unsupervised clustering of the relative expression of EGL-9-repressed and EGL-9-induced genes, in *hif-1*, *egl-9*, *vhl-1*, *swan-1*, and *swan-1;hif-1^P621G^* mutants, as well as *hif-1* animals overexpressing wild type *hif-1* or *hif-1^P621G^* (**Figure 6A, B**). According to EGL-9-repressed gene expression, *vhl-1*, *egl-9*, and *hif-1^P621G^* mutants clustered together, supporting our previous conclusion that canonical signaling represses HIF-1-mediated defense gene induction (**Figure 6A**). In contrast, according to EGL-9-induced gene expression, *egl-9* animals clustered together with *hif-1;[hif-1]* animals, and *vhl-1* animals clustered with *hif-1;[hif-1^P621G^]* animals. *swan-1* animals were an outgroup (**Figure 6B**). However, if *Y65B4BR.1* (whose expression in *swan-1* mutants is very different from *egl-9* mutants, and thus may be an outlier) was excluded, *swan-1* mutants clustered with *egl-9*, *hif-1;[hif-1]*, and *swan-1;hif-1^P621G^* mutants (**Figure S6**). These results support our conclusions that the expression profiles of *egl-9-*repressed genes in *vhl-1* and *egl-9* mutants are similar, while those of *egl-9-*induced genes in *egl-9* and *swan-1* are similar.

### Repression of *egl-9-*induced genes causes enhanced susceptibility

One model to explain our results is that loss of *egl-9* causes enhanced susceptibility to *S. aureus* because it leads to HIF-1-mediated repression of genes that are important for host defense. To test this model, we performed simultaneous RNAi-mediated knockdown of the three most highly repressed genes in *egl-9* mutants, namely *ilys-3*, *Y65B4BR.1*, and *lys-5* (see **Figure 3I**), which when knocked down individually did not lead to enhanced susceptibility [5]. The triple knockdown was sufficient to confer enhanced susceptibility to *S. aureus* (**Figure 5E**), essentially recapitulating the susceptibility phenotype of *egl-9* and *swan-1* mutants. This result therefore supports the notion that repression of host defense genes in *egl-9* mutants is sufficient to cause enhanced susceptibility.

## Discussion

Based on the unexpected result that loss of the prolyl hydroxylase *egl-9* causes enhanced susceptibility to *S. aureus* but enhanced resistance to *P. aeruginosa*, we have found a novel role for HIF in host defense. We find that HIF can modulate the specificity of pathogen-triggered host responses, through activation and repression of host defense gene expression. Most importantly, our results provide a rationale for the existence of both canonical and noncanonical pathways for HIF regulation, which possess distinct biologically significant roles in host defense.

HIF is well known to act as an inducer of hypoxia response genes in many organisms, including *C. elegans*. In contrast, the gene repressive activity of HIF is less well understood. In support of our findings of HIF as a repressor of specific defense genes, two recent studies show that *C. elegans* HIF represses the ferritin-encoding genes *ftn-1* and *ftn-2* during iron starvation [29], [30].

**Figure 7:**
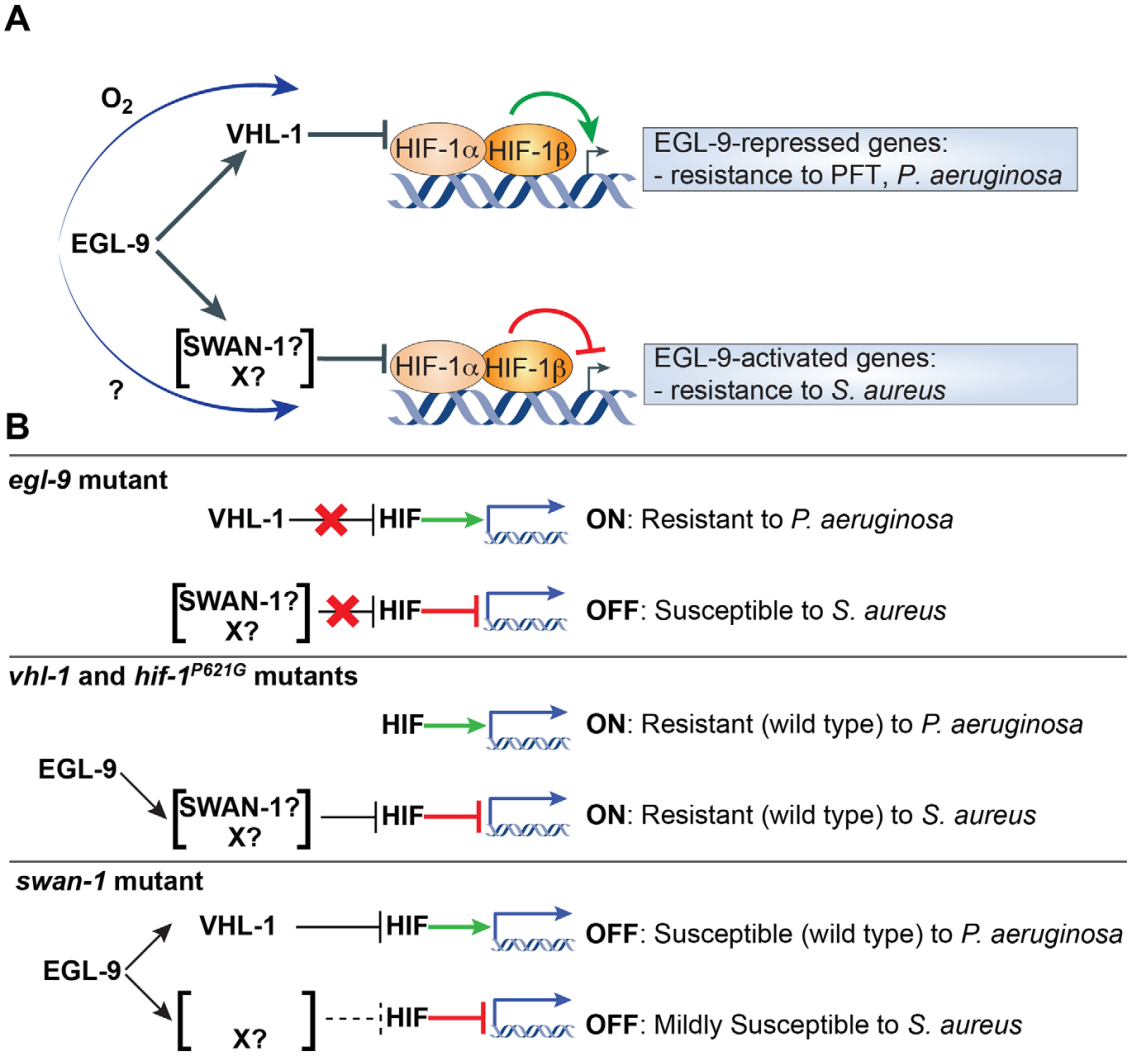
Working model for HIF-1-mediated inflammatory regulation. **A.** Proposed model of noncanonical HIF-1 inhibition lifting HIF-1-mediated repression of host defense genes. Infection by *S. aureus* causes induction of host defense genes. Some of these genes are also induced by HIF, while others are repressed by HIF. Genes induced by HIF include genes that mediate resistance to pore-forming toxins (PFT) and *P. aeruginosa*. Genes that are repressed by HIF include genes that mediate resistance to *S. aureus*. Canonical HIF regulation mediated by VHL-1 requires EGL-9 catalytic activity and controls the gene-inductive activity of HIF. O_2_ is a signal driving HIF inhibition by the canonical pathway. Noncanonical HIF regulation, which is independent of EGL-9 catalytic activity but requires SWAN-1, controls the gene-repressive activity of HIF. The signal(s) that regulate noncanonical signaling are currently not known. **B.** Diagram of predicted and observed phenotypes in mutants defective in canonical or noncanonical HIF signaling.

Both inductive and repressive HIF activities are regulated by EGL-9. Previous studies identified two pathways by which EGL-9 controls HIF, an oxygen-sensitive canonical pathway dependent on EGL-9 catalytic activity and VHL-1 [17], and a noncanonical pathway that partially requires scaffold protein SWAN-1 [18]. However, why EGL-9 would regulate HIF via two parallel pathways remained unclear. We favor a model in which, for a given set of *S. aureus-*induced genes, HIF-1-mediated gene activation is blocked by canonical signaling, while HIF-1-mediated gene repression is lifted by EGL-9 *via* a poorly understood noncanonical pathway that involves SWAN-1 and additional unknown components (**Figure 7A**). Therefore, EGL-9 may represent an important node of host defense modulation, acting to bias host defense gene expression programs in response to contextual cues, such as oxygen or iron availability.

In animals lacking either EGL-9 or VHL-1, HIF-1 enhances the expression of a set of *S. aureus-*induced genes (**Figure 7B**). In *egl-9* mutants, this enhanced expression is offset by concomitant reduced expression of a distinct set of defense genes (resulting in a net susceptibility phenotype), whereas in *vhl-1* mutants it is not. Despite this enhanced expression of host defense genes, *vhl-1* mutants are not more resistant to *S. aureus* than wild type animals. One possible explanation for the lack of effect on host survival in *vhl-1* mutants is that their defense gene overexpression is not large enough to confer resistance. Alternatively, it is possible that additional host defense genes not tested here are concomitantly downregulated in *vhl-1* mutants, producing no net difference in host survival. It is also possible that *vhl-1* mutation has *hif-1*-independent deleterious effects on viability that mask the beneficial effect of defense gene overexpression. As evidence for this, *vhl-1;hif-1* double mutants (which no longer overexpress host defense genes) exhibit a small but discernible enhancement of susceptibility to *S. aureus*. In contrast to these observations, loss of *vhl-1* confers enhanced susceptibility to *P. aeruginosa* cyanide-mediated killing [18].

Despite also causing constitutive overexpression of a set of host defense genes, *egl-9* inactivation conferred hypersusceptibility to *S. aureus*, likely as a result of the repression of important host defense genes. In support of this view, *swan-1* mutants exhibit an overall trend of defense gene repression, as well as increased susceptibility to *S. aureus* (**Figure 7B**). In contrast, *swan-1* mutants exhibit wild-type susceptibility to *P. aeruginosa* [18]. During *S. aureus* infection, the effect of *swan-1* mutation on gene expression and susceptibility is much smaller than that of *egl-9* mutation, suggesting that noncanonical HIF-1 repression may remain partially functional in *swan-1* mutants. Each downregulated defense gene likely contributes incrementally to enhanced susceptibility, as triple knockdown of *ilys-3*, *Y65B4BR.1*, and *lys-5* conferred hypersusceptibility to *S. aureus*, whereas none did so when inhibited individually [5]. The net effect is that *egl-9* mutants display enhanced susceptibility to *S. aureus*.

Our proposed model provides a rationale for the existence of two distinct pathways for HIF-1 inhibition, both of which require EGL-9, since each pathway separately regulates each of two distinct gene-specific activities of HIF-1 (**Figure 7A**). One advantage of this pathway design may be that it allows for integration of multiple signal inputs that regulate the balance of HIF activation and repression activity and, thus, host response specificity, in a context-specific manner (**Figure 7A**). For example, it could allow de-repression of host defense genes downstream of pathogen detection (by the noncanonical branch), while integrating information about oxygen concentration (by the canonical branch), which may be important in the natural setting of *C. elegans*-microbe interactions. The putative signals that modulate the noncanonical branch are not yet known.

In contrast to our findings, upregulation of HIF-1 in *vhl-1* and *egl-9* mutants was previously shown to confer resistance to *B. thuringiensis* and *V. cholerae* pore-forming toxins [22]. Mutation of *egl-9* also confers enhanced resistance to *P. aeruginosa* PAO1 cyanide-mediated killing and enteropathogenic *E. coli* (EPEC) toxin-mediated killing [18], [31], [32]. Additionally, *egl-9* mutations caused enhanced resistance to *P. aeruginosa* PA14 “slow killing” [22], [33]. Consistently, we observed constitutive upregulation of seven out of ten markers of the *P. aeruginosa*-triggered host response [34] in *egl-9* mutants (**Figure S7**). In contrast, in a model for *Burkholderia pseudomallei* pathogenesis *egl-9* mutation did not confer protection [35]. Therefore, the biological functions of EGL-9 vary depending on the nature of the infection, supporting the view that EGL-9/HIF-1 pathways can bias the host response to infection, with different biological effects in different infection scenarios.

In mouse models of inflammation, activation of HIF-1 has different effects depending on the tissue. In myeloid cells, HIF-1 is activated by NF-κB and enhances pro-inflammatory and antimicrobial responses [21], [36]–[39]. In the intestinal epithelium, the role of HIF-1 is less understood. In a recent study, overexpression of HIF-1 protected against colonic inflammation caused by trinitrobenzene sulfonic acid (TNBS) [40]. Additionally, chemical or genetic ablation of EGL-9 homolog PHD1 in intestinal epithelial cells diminished colitis caused by other toxins that disrupt the barrier, such as dextran sodium sulphate (DSS) and *Clostridium difficile* TcdbA and TcdB, suggesting that inhibition of PHD1 causes repression of the inflammatory response [23], [24]. These results mirror our findings in *C. elegans*, where deletion of *egl-9* caused repression of a set of host response genes.

In contrast to the repressive effect of PHD deletion, conditional deletion of *VHL* in the intestinal epithelial cells led to a hyperactive host response, measured as chronic intestinal inflammation and increased susceptibility to DSS; this effect was mediated by HIF1 paralog HIF2 [41]. These results mirror our observations with *C. elegans vhl-1* mutants, which also exhibit constitutive induction of host response genes in the intestinal epithelium, a worm equivalent of chronic molecular inflammation. Thus, the opposing functions of HIF in intestinal gene expression appear to be evolutionarily conserved.

Because of its conserved roles in host defense and inflammation, HIF modulation may be a useful therapeutic approach. Anti-inflammatory therapies are currently under development, which seek to inhibit PHDs to drive HIF upregulation [42]. However, our observations and the cited examples sound a cautionary note against wholesale activation or inhibition of HIF-1, as this may result in beneficial or detrimental effects on host intestinal homeostasis depending on the physiological context. In certain instances, such as *C. difficile*-triggered inflammation [24], HIF augmentation may result in beneficial inhibition of damage. In other infections, HIF-1 augmentation may prevent a beneficial host response, as illustrated by the present studies. Further work is required to elucidate the precise mechanisms by which HIF attenuates inflammation and the clinical scenarios where pharmacologic manipulation of HIF may have beneficial effects.

On the basis of the accumulated evidence, we propose that mammalian and nematode HIF may have important roles as repressors of the host response in intestinal epithelial cells, and that noncanonical signaling may be a specific mechanism of control of HIF-mediated gene repression. Although mounting evidence supports a role for HIF as a repressor of gene expression in nematodes and mammals [29], [43]–[49], little is known about the physiological consequences of this repression in the context of intestinal host-microbiota interactions. In humans, HIF-1 accumulates during a wide range of infections [20] as well as chronic intestinal inflammation [50]. Additionally, DDB1- and CUL4-associated factor 7 (DCAF7), the human homolog of SWAN-1, is differentially regulated in the intestinal epithelium and in blood from patients suffering from Crohn's disease or ulcerative colitis, two forms of inflammatory bowel disease [51]–[57]. Thus, HIF signaling components are likely to be expressed at infection sites, and it is tempting to speculate that noncanonical HIF-1 signaling may be functionally relevant in intestinal inflammation in humans, as in *C. elegans*.

## Materials and Methods

### Strains

*C. elegans* was grown on nematode-growth media (NGM) plates seeded with *E. coli* OP50-1 at 15–20°C according to standard procedures [58]. *C. elegans* strains used in this study are detailed in Table S1a. Bacterial strains are detailed in Table S1b.

### Infection assays

*S. aureus killing assays.* Assays were performed as described [12]. Briefly, NCTC8325 was grown overnight in tryptic soy broth (TSB, BD, Sparks, MD) with 10 µg/ml nalidixic acid (Sigma). 10 µl of overnight cultures were seeded on 35 mm tryptic soy agar (TSA, BD, Sparks, MD) plates with 10 µg/ml nalidixic acid. To sterilize worms before use in killing assays, so that strains with potential differences in fertility or egg-laying behavior could be directly compared, *cdc-25.1* RNAi was carried out by feeding L4 animals for 24 h at 15°C. Animals that exhibit an Emb phenotype [59] were selected for further analysis. A total of 25–35 late-L4 stage hermaphrodites were transferred to each of three replicate plates per strain. Animals that died because of a bursting vulva or crawled off the agar were censored. Experiments were performed at least twice. All infection assays were conducted at 25°C, 70% relative humidity. Animals were scored as alive or dead by gentle prodding with a platinum wire. Kaplan-Meier statistical analyses were performed using Prism 5 software (GraphPad). Survival data were compared as described using the log-rank test. Data are represented as median survival (MS), as defined by Kaplan-Meier analysis, or Time to 50% Death – 50 (LT_50_), as defined by nonlinear regression, when MS values were skewed by having a small number of timepoints, N (total number of animals/censored), and *p* value. A *p*-value<0.05 was considered significantly different from control.

### Quantitative RT-PCR (qRT-PCR) analysis

Animals were treated essentially as described for killing assays described above, with the following modifications. For *S. aureus* infection assays, infected samples were compared with parallel samples feeding on *E. coli* OP50, heat-killed by 30 min incubation at 95°C, plated on the same TSA medium. Total RNA was extracted using TRI Reagent (MRC), and reverse transcribed using the Superscript III kit (Invitrogen). cDNA was subjected to qRT-PCR analysis using SYBR green detection (Bio-Rad) on an iCycler machine (Eppendorf). Primers for qRT-PCR were usually designed to span an intron, using the Primer-BLAST tool of the National Center for Biotechnology Information of the National Institutes of Health (http://www.ncbi.nlm.nih.gov/tools/primer-blast/) and checked for specificity against the *C. elegans* genome. All values are normalized against the control gene *snb-1*, which did not vary under conditions being tested. Fold change was calculated using the Pfaffl method [60]. Primer sequences are available upon request. Two-sample, two-tailed *t* test statistical analyses were performed to evaluate differences among pooled ΔCt values according to Pfaffl [60] and using Numbers (Apple). A *P* value≤0.05 was considered significant.

### RNAi knockdown

*Triple RNAi.* Enhanced RNAi *eri-1(mg366)* mutants were propagated at 15°C. RNAi of selected genes was carried out in triplicate using bacterial feeding RNAi [61]. Gravid adults were transferred to RNAi plates containing dsRNA-expressing HT115 against *lys-5*, *ilys-3*, and *Y65B4BR.1* at a ratio of 1∶1∶1, incubated at 15°C for 48 h and then 25°C for 24 h to induce sterility in the progeny, and then transferred to NCTC8325-seeded killing assay plates. RNAi clones were obtained from the Ahringer library and sequences were confirmed [61].

### Epifluorescence microscopy

Animals were mounted on glass slides with 2% agarose pads, anesthetized with 30 mM NaN_3_, and immediately used for imaging. Exposure times were set for the most highly expressed condition and kept constant throughout each experiment. Images were acquired using a Zeiss AXIO Imager Z1 microscope with a Zeiss AxioCam HRm camera and Axiovision 4.6 (Zeiss) software. Image cropping and minimal manipulation were performed using Photoshop (Adobe). Quantification of GFP signal was performed using OpenLab (Improvision Corp.) from equally exposed micrographs, by selecting the posterior third of the intestine and computing mean pixel intensity for the whole area for each animal.

### List of genes

**WormBase ID, Public Name;** WBGene00016017, C23G10.11; WBGene00016923, C54F6.5; WBGene00015052, *clec-52*; WBGene00014046, *clec-60*; WBGene00021582, *clec-71*; WBGene00000528, *clh-1*; WBGene00000782, *cpr-2*; WBGene00020386, *cyp-34A4*; WBGene00001178, *egl-9*; WBGene00001366, *exc-5*; WBGene00017673, F21F3.3; WBGene00018267, F41C3.1; WBGene00018731, F53A9.8; WBGene00001477, *fmo-2*; WBGene00001851, *hif-1*; WBGene00016670, *ilys-3*; WBGene00002094, *ins-11*; WBGene00003094, *lys-5*; WBGene00009977, *swan-1*; WBGene00006611, *tre-5*; WBGene00006922, *vhl-1*; WBGene00022040, Y65B4BR.1

## Supporting Information

Figure S1 **Tissue-specific expression of** ***egl-9*** **.** **A.** CX8628 (*egl-9* mutant expressing neuronal promoter-*egl-9*), CX8756 (*egl-9* mutant expressing *egl-9* promoter-*egl-9*), **B.** CX9778 (*egl-9* mutant expressing epidermal promoter-*egl-9*), and CX8630 (*egl-9* mutant expressing muscle promoter-*egl-9*) animals are hypersusceptible to *S. aureus*, indicating that the transgenes were unable to rescue the survival defect in the *egl-9(sa307)* background, despite being functional for behavioral and egg-laying rescues [1]. This may imply that the *egl-9* promoter used in the rescuing construct lacks regulatory sequences that are essential for rescue of the immunity defect. Accordingly, we observed little GFP expression in the intestine for this construct (not shown). Results are representative of two independent trials, performed in triplicate. N≥100.
(TIF)

Figure S2 ***hif-1*** **is dispensable for induction of the** ***C. elegans*** **host response to** ***S. aureus*** **.** **A.** *hif-1(ia4)* and wild type animals were fed *E. coli* or infected with *S. aureus* for 8 h and gene expression was measured by qRT-PCR. Some genes were slightly upregulated (*hif-1-*repressed genes) and *lys-5* was downregulated in uninfected *hif-1(-)* animals. Values were normalized to wild type animals. *, *p*≤0.05 (compared with wild type by two-sample *t* test). **B.** Marker gene induction in *hif-1(ia4)* animals compared with wild type. Values are normalized to uninfected controls of each genotype. n.s., not significant. **C.** *egl-9* and *hif-1* were slightly induced in wild type animals by 8 h infection with *S. aureus*. Data are means of 2–3 independent biological replicates, error bars are SEM. *, *p*≤0.05 (compared with wild type by two-sample *t* test).
(TIF)

Figure S3 ***egl-9, vhl-1*** **mutants exhibit increased** ***clec-60::*** **GFP expression in the intestinal epithelium.** GFP signal was quantified from micrographs at equal exposures, selecting the posterior third of the intestine and computing mean pixel intensity in the selected area and expressed in arbitrary units (a.u.). Horizontal bars represent the population median. *, *p*<0.05 (compared with wild type by Kruskal-Wallis test with Dunn's multiple comparison *post hoc* test).
(TIF)

Figure S4 ***egl-9(sa307)*** **mutants ectopically express** ***Pclec-60::gfp*** **in the excretory cell.** Expression was very low compared to the intestine and not sufficient to account for *clec-60* increased expression in *egl-9* mutants by qRT-PCR.
(TIF)

Figure S5 **Gene expression measured by qRT-PCR in uninfected** ***swan-1(ok267)*** **,** ***swan-1*** **;** ***[hif-1^P621G^]*** **, and** ***egl-9(sa307)*** **mutants, normalized to wild type.** *egl-9(sa307)* data from Figure 3G, 3H and 3I are included for comparison. Data are means of 2–3 independent biological replicates, error bars are SEM.
(TIF)

Figure S6 ***egl-9*** **,** ***swan-1, and swan-1;[hif-1^P621G^]*** **mutants cluster by** ***egl-9-*** **induced gene expression.** Non-hierarchical cluster analysis of *egl-9-*repressed gene expression changes in infected *hif-1(ia4)*, *swan-1(ok267)*, *swan-1(ok267);[hif-1^P621G^]*, *egl-9(sa307)*, *vhl-1(ok161)*, *hif-1(ia4);[hif-1^P621G^]*, and *hif-1;[hif-1]* animals normalized to wild type, excluding *Y65B4BR.1*. Blue indicates downregulation, red indicates upregulation. Color intensity reflects magnitude of change; darker colors correspond to larger changes.
(TIF)

Figure S7 ***egl-9*** **mutants constitutively overexpress PA14-induced genes.** Expression of ten PA14-induced genes [2] was measured by qRT-PCR in *egl-9(sa307)* mutant and wild type animals. Data are means of two independent biological replicates, normalized to wild type. Error bars are SEM.
(TIF)

Table S1 **List of strains used in this study.**
(DOCX)

## References

[1] Oeckinghaus A, Hayden MS, Ghosh S (2011). Crosstalk in NF-κB signaling pathways.. Nat Immunol.

[2] Amit I, Garber M, Chevrier N, Leite AP, Donner Y (2009). Unbiased reconstruction of a mammalian transcriptional network mediating pathogen responses.. Science.

[3] Ronald PC, Beutler B (2010). Plant and animal sensors of conserved microbial signatures.. Science.

[4] Irazoqui JE, Urbach JM, Ausubel FM (2010). Evolution of host innate defence: insights from Caenorhabditis elegans and primitive invertebrates.. Nat Rev Immunol.

[5] Irazoqui JE, Troemel ER, Feinbaum RL, Luhachack LG, Cezairliyan BO (2010). Distinct pathogenesis and host responses during infection of C. elegans by P. aeruginosa and S. aureus.. PLoS Pathog.

[6] Zugasti O, Ewbank JJ (2009). Neuroimmune regulation of antimicrobial peptide expression by a noncanonical TGF-beta signaling pathway in Caenorhabditis elegans epidermis.. Nat Immunol.

[7] Tenor JL, Aballay A (2008). A conserved Toll-like receptor is required for Caenorhabditis elegans innate immunity.. EMBO Rep.

[8] Kurz CL, Ewbank JJ (2003). Caenorhabditis elegans: an emerging genetic model for the study of innate immunity.. Nat Rev Genet.

[9] Wong D, Bazopoulou D, Pujol N, Tavernarakis N, Ewbank JJ (2007). Genome-wide investigation reveals pathogen-specific and shared signatures in the response of Caenorhabditis elegans to infection.. Genome Biol.

[10] Nicholas HR, Hodgkin J (2004). The ERK MAP kinase cascade mediates tail swelling and a protective response to rectal infection in C. elegans.. Curr Biol.

[11] Kim DH, Feinbaum R, Alloing G, Emerson FE, Garsin DA (2002). A conserved p38 MAP kinase pathway in Caenorhabditis elegans innate immunity.. Science.

[12] Irazoqui JE, Ng A, Xavier RJ, Ausubel FM (2008). Role for beta-catenin and HOX transcription factors in Caenorhabditis elegans and mammalian host epithelial-pathogen interactions.. Proc Natl Acad Sci USA.

[13] Semenza GL (2001). HIF-1, O(2), and the 3 PHDs: how animal cells signal hypoxia to the nucleus.. Cell.

[14] Powell-Coffman JA (2010). Hypoxia signaling and resistance in C. elegans.. Trends Endocrinol Metab.

[15] Jaakkola P, Mole DR, Tian YM, Wilson MI, Gielbert J (2001). Targeting of HIF-alpha to the von Hippel-Lindau ubiquitylation complex by O2-regulated prolyl hydroxylation.. Science.

[16] Ivan M, Kondo K, Yang H, Kim W, Valiando J (2001). HIFalpha targeted for VHL-mediated destruction by proline hydroxylation: implications for O2 sensing.. Science.

[17] Epstein AC, Gleadle JM, McNeill LA, Hewitson KS, O'Rourke J (2001). C. elegans EGL-9 and mammalian homologs define a family of dioxygenases that regulate HIF by prolyl hydroxylation.. Cell.

[18] Shao Z, Zhang Y, Ye Q, Saldanha JN, Powell-Coffman JA (2010). C. elegans SWAN-1 Binds to EGL-9 and Regulates HIF-1-Mediated Resistance to the Bacterial Pathogen Pseudomonas aeruginosa PAO1.. PLoS Pathog.

[19] Shao Z, Zhang Y, Powell-Coffman JA (2009). Two distinct roles for EGL-9 in the regulation of HIF-1-mediated gene expression in Caenorhabditis elegans.. Genetics.

[20] Werth N, Beerlage C, Rosenberger C, Yazdi AS, Edelmann M (2010). Activation of hypoxia inducible factor 1 is a general phenomenon in infections with human pathogens.. PLoS ONE.

[21] Peyssonnaux C, Datta V, Cramer T, Doedens A, Theodorakis EA (2005). HIF-1alpha expression regulates the bactericidal capacity of phagocytes.. J Clin Invest.

[22] Bellier A, Chen C-S, Kao C-Y, Cinar HN, Aroian RV (2009). Hypoxia and the hypoxic response pathway protect against pore-forming toxins in C. elegans.. PLoS Pathog.

[23] Tambuwala MM, Cummins EP, Lenihan CR, Kiss J, Stauch M (2010). Loss of prolyl hydroxylase-1 protects against colitis through reduced epithelial cell apoptosis and increased barrier function.. Gastroenterology.

[24] Hirota SA, Fines K, Ng J, Traboulsi D, Lee J (2010). Hypoxia-inducible factor signaling provides protection in Clostridium difficile-induced intestinal injury.. Gastroenterology.

[25] Sifri CD, Begun J, Ausubel FM, Calderwood SB (2003). Caenorhabditis elegans as a model host for Staphylococcus aureus pathogenesis.. Infect Immun.

[26] Nygaard TK, DeLeo FR, Voyich JM (2008). Community-associated methicillin-resistant Staphylococcus aureus skin infections: advances toward identifying the key virulence factors.. Curr Opin Infect Dis.

[27] Chen D, Thomas EL, Kapahi P (2009). HIF-1 modulates dietary restriction-mediated lifespan extension via IRE-1 in Caenorhabditis elegans.. PLoS Genet.

[28] Lee S-J, Hwang AB, Kenyon C (2010). Inhibition of respiration extends C. elegans life span via reactive oxygen species that increase HIF-1 activity.. Curr Biol.

[29] Romney SJ, Newman BS, Thacker C, Leibold EA (2011). HIF-1 Regulates Iron Homeostasis in Caenorhabditis elegans by Activation and Inhibition of Genes Involved in Iron Uptake and Storage.. PLoS Genet.

[30] Ackerman D, Gems D (2012). Insulin/IGF-1 and Hypoxia Signaling Act in Concert to Regulate Iron Homeostasis in Caenorhabditis elegans.. PLoS Genet.

[31] Anyanful A, Dolan-Livengood JM, Lewis T, Sheth S, Dezalia MN (2005). Paralysis and killing of Caenorhabditis elegans by enteropathogenic Escherichia coli requires the bacterial tryptophanase gene.. Mol Microbiol.

[32] Darby C, Cosma CL, Thomas JH, Manoil C (1999). Lethal paralysis of Caenorhabditis elegans by Pseudomonas aeruginosa.. Proc Natl Acad Sci USA.

[33] Tan M-W, Mahajan-Miklos S, Ausubel FM (1999). Killing of Caenorhabditis elegans by Pseudomonas aeruginosa used to model mammalian bacterial pathogenesis.. Proc Natl Acad Sci USA.

[34] Troemel ER, Chu SW, Reinke V, Lee SS, Ausubel FM (2006). p38 MAPK regulates expression of immune response genes and contributes to longevity in C. elegans.. PLoS Genet.

[35] Gan Y-H, Chua KL, Chua HH, Liu B, Hii CS (2002). Characterization of Burkholderia pseudomallei infection and identification of novel virulence factors using a Caenorhabditis elegans host system.. Mol Microbiol.

[36] Cramer T, Yamanishi Y, Clausen BE, Förster I, Pawlinski R (2003). HIF-1alpha is essential for myeloid cell-mediated inflammation.. Cell.

[37] Peyssonnaux C, Cejudo-Martin P, Doedens A, Zinkernagel AS, Johnson RS (2007). Cutting edge: Essential role of hypoxia inducible factor-1alpha in development of lipopolysaccharide-induced sepsis.. J Immunol.

[38] Rius J, Guma M, Schachtrup C, Akassoglou K, Zinkernagel AS (2008). NF-kappaB links innate immunity to the hypoxic response through transcriptional regulation of HIF-1alpha.. Nature.

[39] Zinkernagel AS, Peyssonnaux C, Johnson RS, Nizet V (2008). Pharmacologic augmentation of hypoxia-inducible factor-1alpha with mimosine boosts the bactericidal capacity of phagocytes.. J Infect Dis.

[40] Karhausen J, Furuta GT, Tomaszewski JE, Johnson RS, Colgan SP (2004). Epithelial hypoxia-inducible factor-1 is protective in murine experimental colitis.. J Clin Invest.

[41] Shah YM, Ito S, Morimura K, Chen C, Yim S-H (2008). Hypoxia-inducible factor augments experimental colitis through an MIF-dependent inflammatory signaling cascade.. Gastroenterology.

[42] Robinson A, Keely S, Karhausen J, Gerich ME, Furuta GT (2008). Mucosal protection by hypoxia-inducible factor prolyl hydroxylase inhibition.. Gastroenterology.

[43] Mole DR, Blancher C, Copley RR, Pollard PJ, Gleadle JM (2009). Genome-wide association of hypoxia-inducible factor (HIF)-1alpha and HIF-2alpha DNA binding with expression profiling of hypoxia-inducible transcripts.. J Biol Chem.

[44] Peyssonnaux C, Zinkernagel AS, Schuepbach RA, Rankin E, Vaulont S (2007). Regulation of iron homeostasis by the hypoxia-inducible transcription factors (HIFs).. J Clin Invest.

[45] Ibla JC, Khoury J, Kong T, Robinson A, Colgan SP (2006). Transcriptional repression of Na-K-2Cl cotransporter NKCC1 by hypoxia-inducible factor-1.. Am J Physiol Cell Physiol.

[46] Morote-Garcia JC, Rosenberger P, Kuhlicke J, Eltzschig HK (2008). HIF-1-dependent repression of adenosine kinase attenuates hypoxia-induced vascular leak.. Blood.

[47] Morote-Garcia JC, Rosenberger P, Nivillac NMI, Coe IR, Eltzschig HK (2009). Hypoxia-inducible factor-dependent repression of equilibrative nucleoside transporter 2 attenuates mucosal inflammation during intestinal hypoxia.. Gastroenterology.

[48] Chen K-F, Lai Y-Y, Sun HS, Tsai S-J (2005). Transcriptional repression of human cad gene by hypoxia inducible factor-1alpha.. Nucleic Acids Res.

[49] Eltzschig HK, Abdulla P, Hoffman E, Hamilton KE, Daniels D (2005). HIF-1-dependent repression of equilibrative nucleoside transporter (ENT) in hypoxia.. J Exp Med.

[50] Colgan SP, Taylor CT (2010). Hypoxia: an alarm signal during intestinal inflammation.. Nat Rev Gastroenterol Hepatol.

[51] Galamb O, Sipos F, Solymosi N, Spisák S, Krenács T (2008). Diagnostic mRNA expression patterns of inflamed, benign, and malignant colorectal biopsy specimen and their correlation with peripheral blood results.. Cancer Epidemiol Biomarkers Prev.

[52] Csillag C, Borup R, Olsen J, Nielsen FC, Nielsen OH (2007). Treatment response and colonic gene expression in patients with Crohn's disease.. Scand J Gastroenterol.

[53] Arijs I, De Hertogh G, Lemaire K, Quintens R, Van Lommel L (2009). Mucosal gene expression of antimicrobial peptides in inflammatory bowel disease before and after first infliximab treatment.. PLoS ONE.

[54] Costello CM, Mah N, Häsler R, Rosenstiel P, Waetzig GH (2005). Dissection of the inflammatory bowel disease transcriptome using genome-wide cDNA microarrays.. PLoS Med.

[55] Burczynski ME, Peterson RL, Twine NC, Zuberek KA, Brodeur BJ (2006). Molecular classification of Crohn's disease and ulcerative colitis patients using transcriptional profiles in peripheral blood mononuclear cells.. J Mol Diagn.

[56] Watanabe T, Kobunai T, Toda E, Kanazawa T, Kazama Y (2007). Gene expression signature and the prediction of ulcerative colitis-associated colorectal cancer by DNA microarray.. Clin Cancer Res.

[57] Ahrens R, Waddell A, Seidu L, Blanchard C, Carey R (2008). Intestinal macrophage/epithelial cell-derived CCL11/eotaxin-1 mediates eosinophil recruitment and function in pediatric ulcerative colitis.. J Immunol.

[58] Powell JR, Ausubel FM (2008). Models of Caenorhabditis elegans infection by bacterial and fungal pathogens.. Method Mol Biol.

[59] Evans EA, Chen WC, Tan M-W (2008). The DAF-2 insulin-like signaling pathway independently regulates aging and immunity in C. elegans.. Aging Cell.

[60] Pfaffl MW (2001). A new mathematical model for relative quantification in real-time RT-PCR.. Nucleic Acids Res.

[61] Kamath RS, Ahringer J (2003). Genome-wide RNAi screening in Caenorhabditis elegans.. Methods.

