# Supplementary figures and images for: EGL-9 Controls C. elegans Host Defense Specificity through Prolyl Hydroxylation-Dependent and -Independent HIF-1 Pathways
Source: PLoS Pathog. 2012 Jul 5;8(7):e1002798. doi: 10.1371/journal.ppat.1002798 (PMC3390412; doi:10.1371/journal.ppat.1002798)

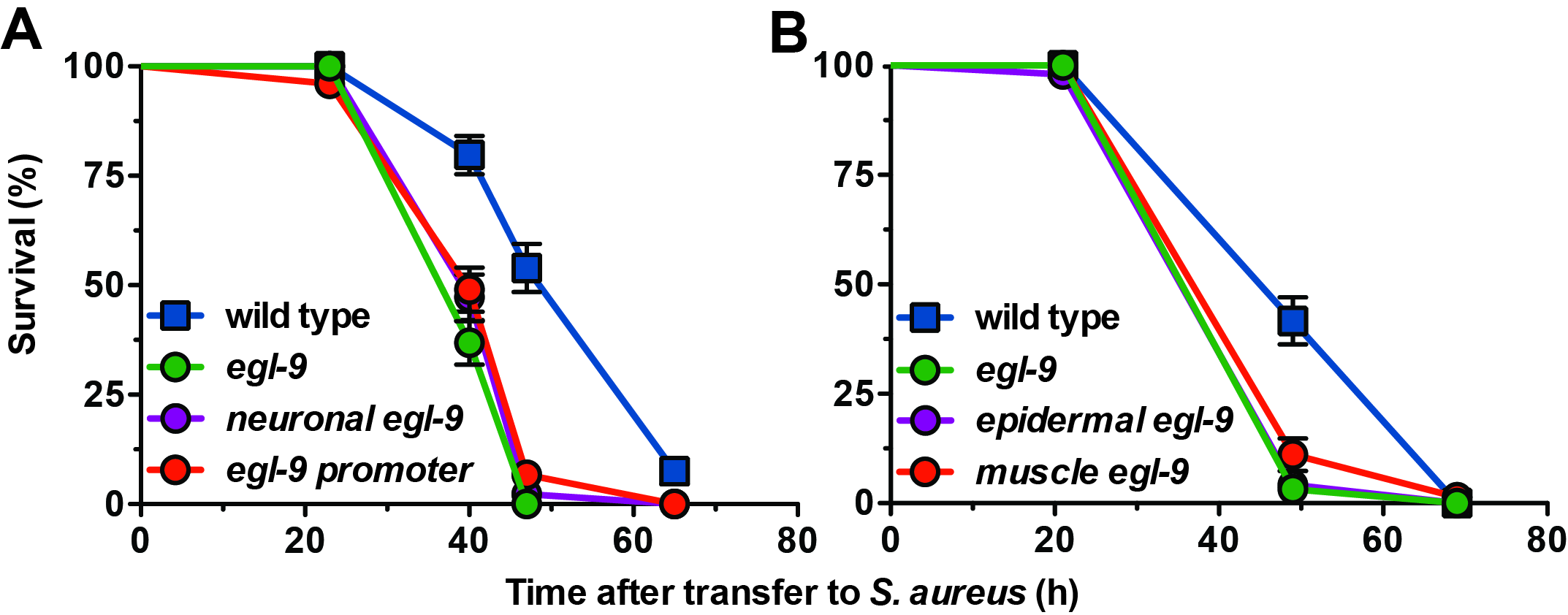

Supplement: Figure S1 — Tissue-specific expression of egl-9 . A. CX8628 (egl-9 mutant expressing neuronal promoter-egl-9), CX8756 (egl-9 mutant expressing egl-9 promoter-egl-9), B. CX9778 (egl-9 mutant expressing epidermal promoter-egl-9), and CX8630 (egl-9 mutant expressing muscle promoter-egl-9) animals are hypersusceptible to S. aureus, indicating that the transgenes were unable to rescue the survival defect in the egl-9(sa307) background, despite being functional for behavioral and egg-laying rescues [1]. This may imply that the egl-9 promoter used in the rescuing construct lacks regulatory sequences that are essential for rescue of the immunity defect. Accordingly, we observed little GFP expression in the intestine for this construct (not shown). Results are representative of two independent trials, performed in triplicate. N≥100. (TIF) [file ppat.1002798.s001.tif]

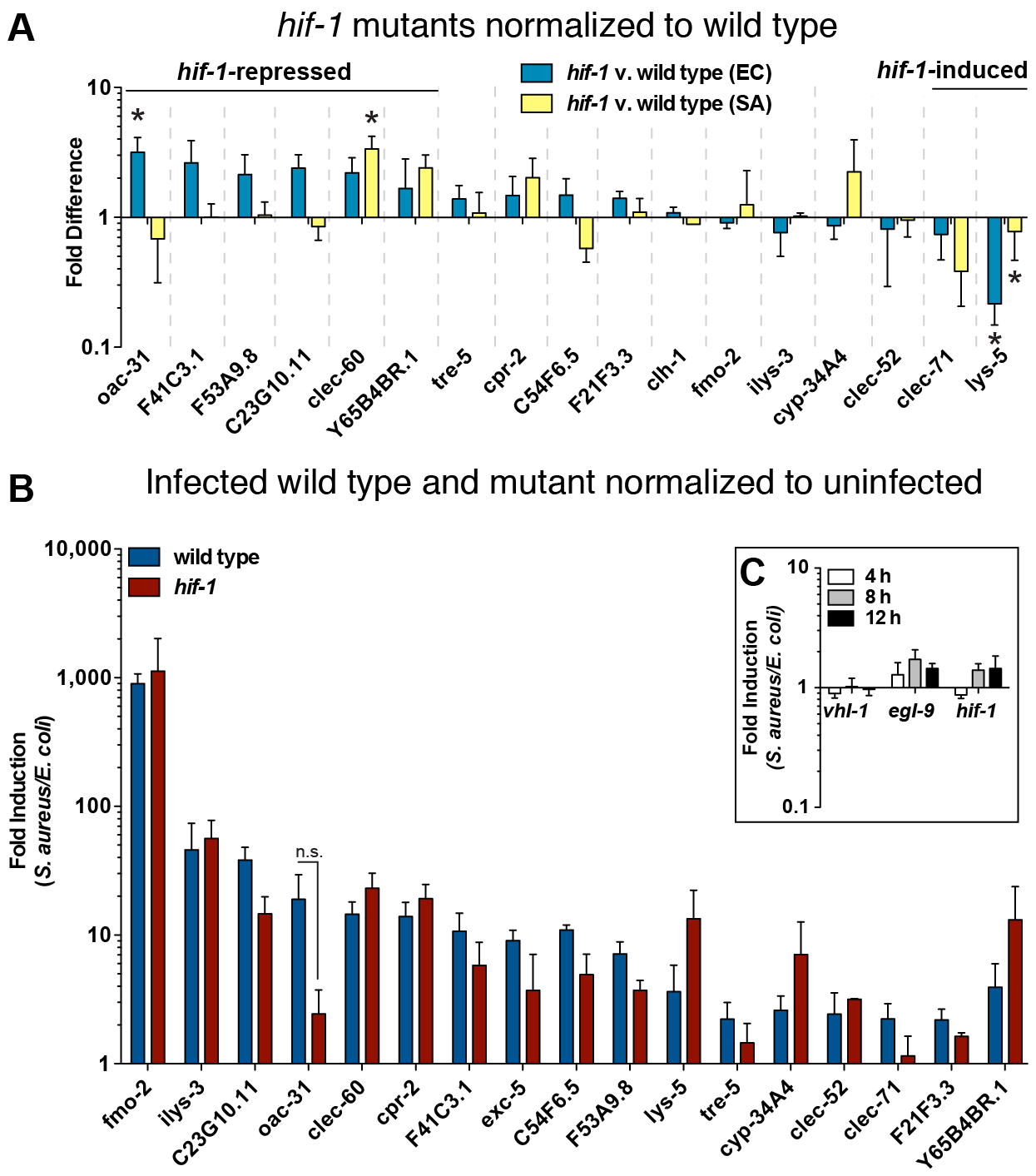

Supplement: Figure S2 — hif-1 is dispensable for induction of the C. elegans host response to S. aureus . A. hif-1(ia4) and wild type animals were fed E. coli or infected with S. aureus for 8 h and gene expression was measured by qRT-PCR. Some genes were slightly upregulated (hif-1-repressed genes) and lys-5 was downregulated in uninfected hif-1(-) animals. Values were normalized to wild type animals. *, p≤0.05 (compared with wild type by two-sample t test). B. Marker gene induction in hif-1(ia4) animals compared with wild type. Values are normalized to uninfected controls of each genotype. n.s., not significant. C. egl-9 and hif-1 were slightly induced in wild type animals by 8 h infection with S. aureus. Data are means of 2–3 independent biological replicates, error bars are SEM. *, p≤0.05 (compared with wild type by two-sample t test). (TIF) [file ppat.1002798.s002.tif]

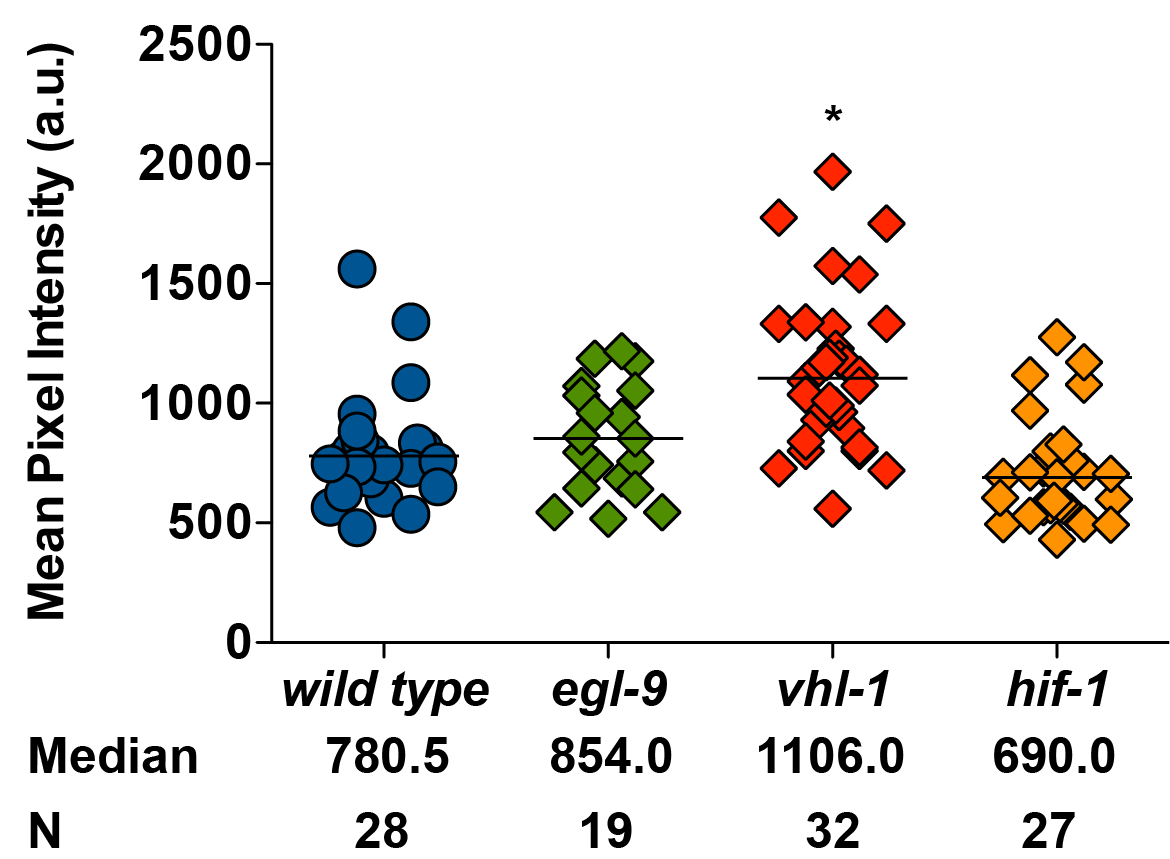

Supplement: Figure S3 — egl-9, vhl-1 mutants exhibit increased clec-60:: GFP expression in the intestinal epithelium. GFP signal was quantified from micrographs at equal exposures, selecting the posterior third of the intestine and computing mean pixel intensity in the selected area and expressed in arbitrary units (a.u.). Horizontal bars represent the population median. *, p<0.05 (compared with wild type by Kruskal-Wallis test with Dunn's multiple comparison post hoc test). (TIF) [file ppat.1002798.s003.tif]

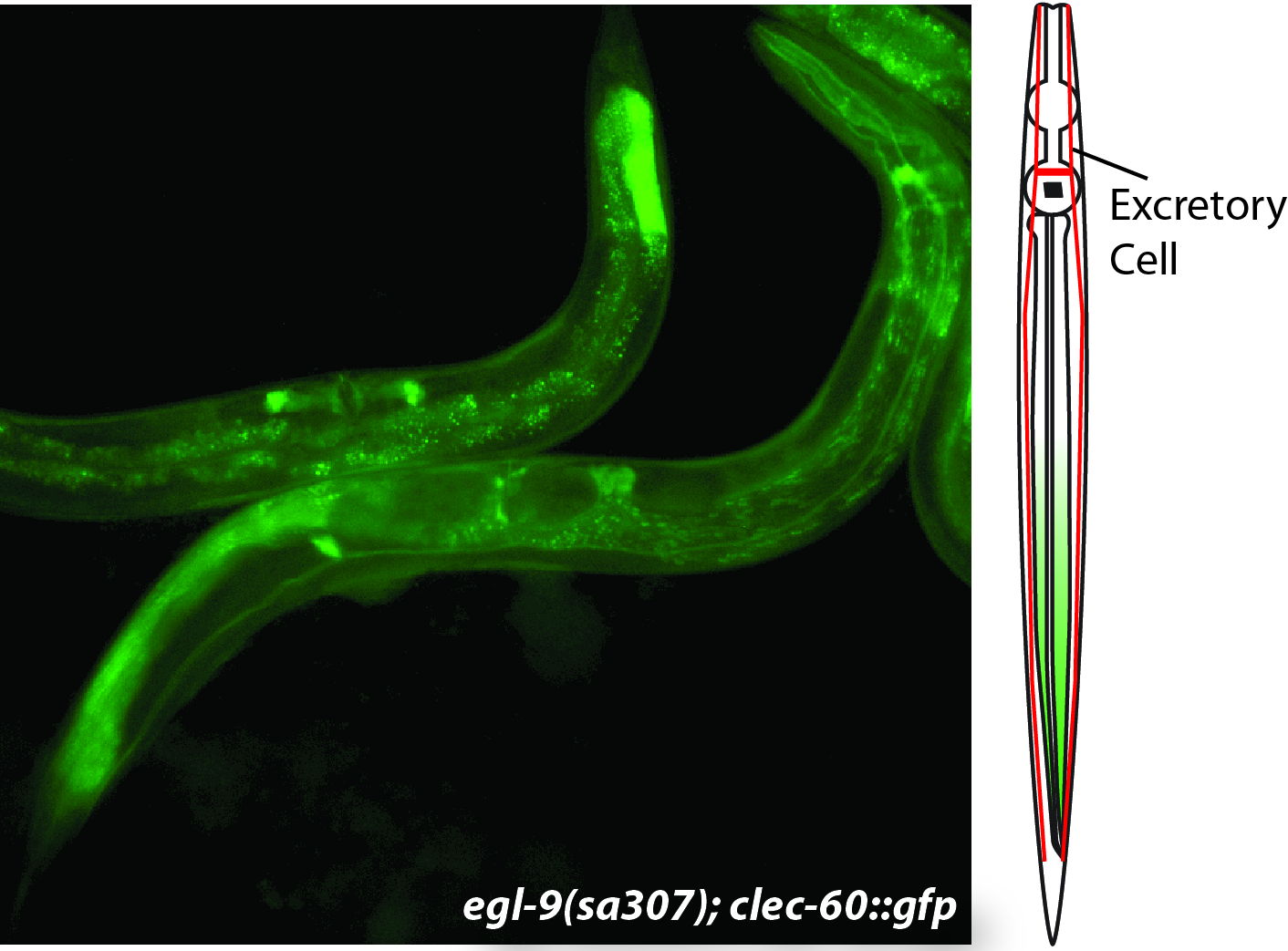

Supplement: Figure S4 — egl-9(sa307) mutants ectopically express Pclec-60::gfp in the excretory cell. Expression was very low compared to the intestine and not sufficient to account for clec-60 increased expression in egl-9 mutants by qRT-PCR. (TIF) [file ppat.1002798.s004.tif]

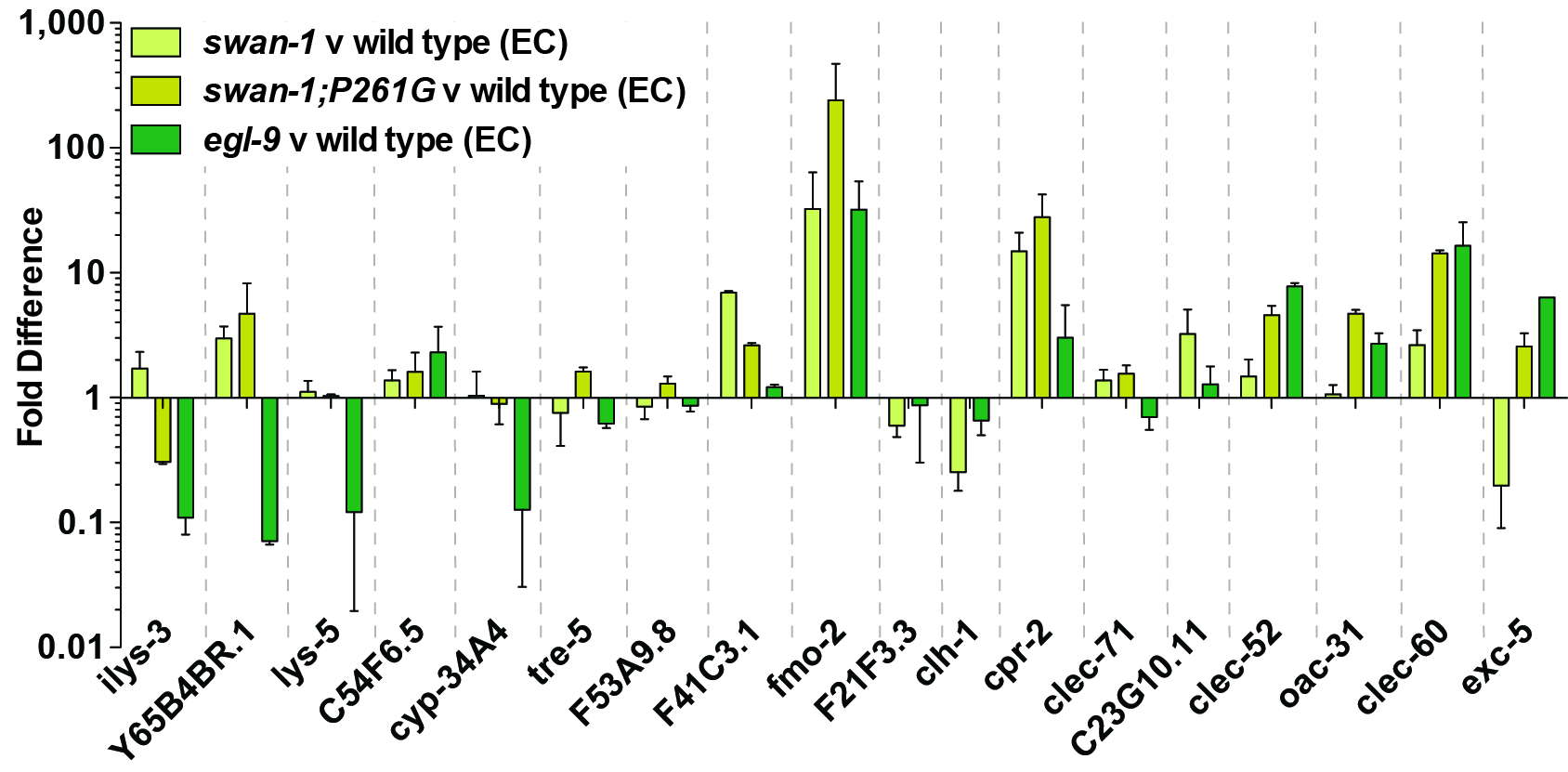

Supplement: Figure S5 — Gene expression measured by qRT-PCR in uninfected swan-1(ok267) , swan-1 ; [hif-1P621G] , and egl-9(sa307) mutants, normalized to wild type. egl-9(sa307) data from Figure 3G, 3H and 3I are included for comparison. Data are means of 2–3 independent biological replicates, error bars are SEM. (TIF) [file ppat.1002798.s005.tif]

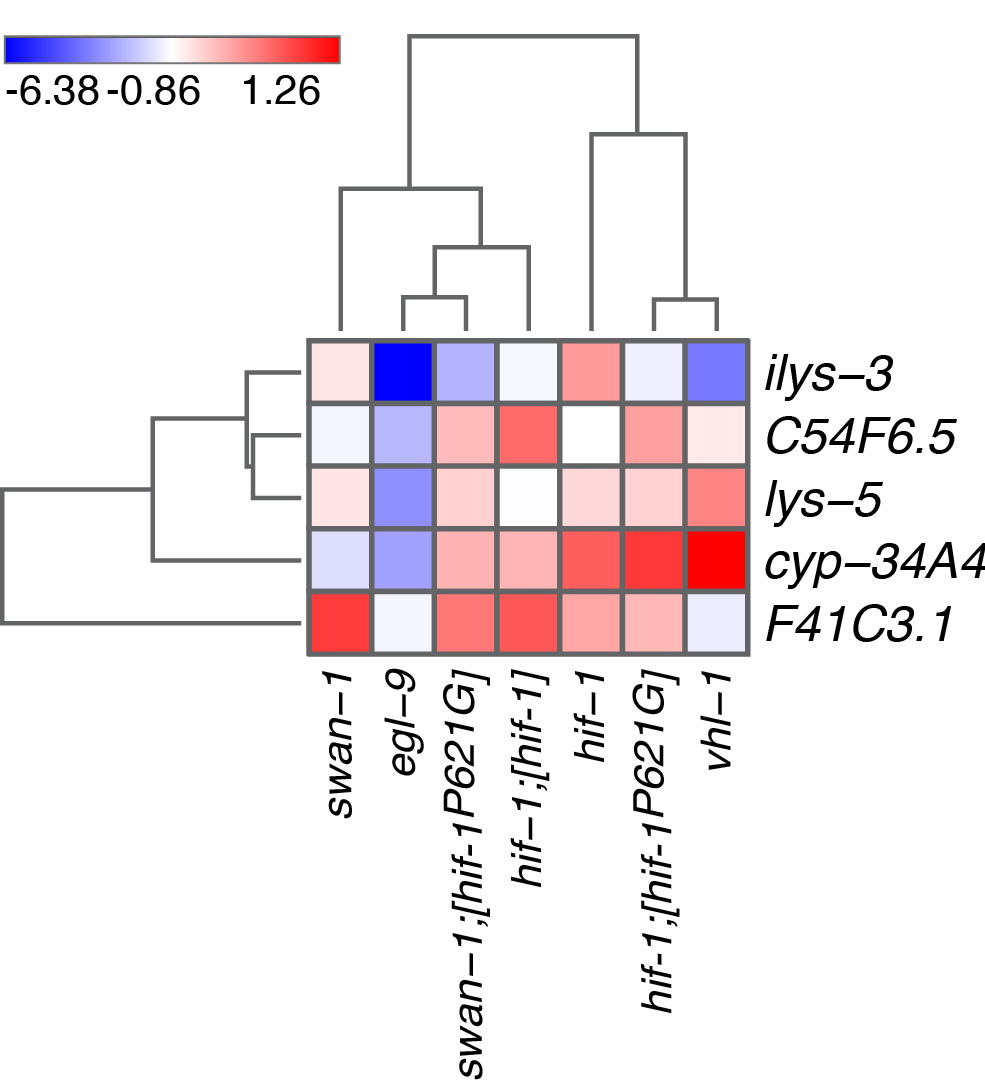

Supplement: Figure S6 — egl-9 , swan-1, and swan-1;[hif-1P621G] mutants cluster by egl-9- induced gene expression. Non-hierarchical cluster analysis of egl-9-repressed gene expression changes in infected hif-1(ia4), swan-1(ok267), swan-1(ok267);[hif-1P621G], egl-9(sa307), vhl-1(ok161), hif-1(ia4);[hif-1P621G], and hif-1;[hif-1] animals normalized to wild type, excluding Y65B4BR.1. Blue indicates downregulation, red indicates upregulation. Color intensity reflects magnitude of change; darker colors correspond to larger changes. (TIF) [file ppat.1002798.s006.tif]

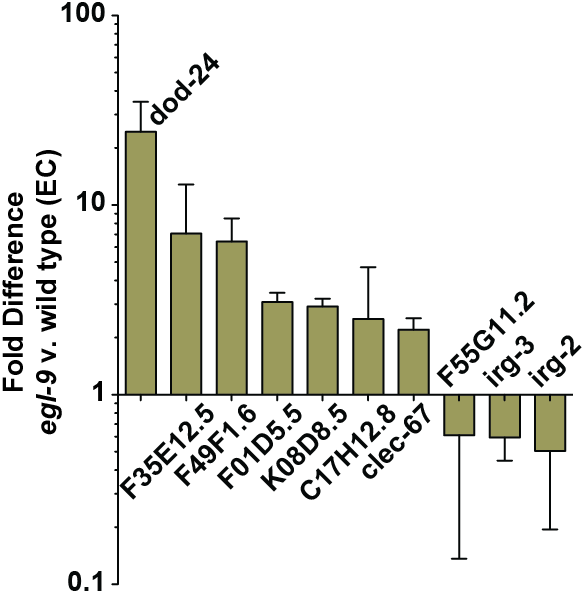

Supplement: Figure S7 — egl-9 mutants constitutively overexpress PA14-induced genes. Expression of ten PA14-induced genes [2] was measured by qRT-PCR in egl-9(sa307) mutant and wild type animals. Data are means of two independent biological replicates, normalized to wild type. Error bars are SEM. (TIF) [file ppat.1002798.s007.tif]
