# Supplementary material for: EGL-9 Controls C. elegans Host Defense Specificity through Prolyl Hydroxylation-Dependent and -Independent HIF-1 Pathways
Source: PLoS Pathog. 2012 Jul 5;8(7):e1002798. doi: 10.1371/journal.ppat.1002798 (PMC3390412; doi:10.1371/journal.ppat.1002798)
Supplement: Table S1 — List of strains used in this study. (DOCX) [file ppat.1002798.s008.docx]

**Supporting Online Material**

**Table S1. Strains.**

**S1a. *C. elegans* strains.**

| Name | Relevant genotype | Source |
| --- | --- | --- |
| AU0281 | agEx99[myo-2::mCherry,ilys-3::gfp] | [3] |
| AU185 | agIs26 [myo-2::mCherry,clec-60::gfp] | This work |
| CB5602 | vhl-1(ok161) X. | Stock center (CGC) |
| CB6088 | egl-9(sa307),hif-1(ia4) | Stock center (CGC) |
| CB6090 | hif-1(ia4);vhl-1(ok161) | Stock center (CGC) |
| CX8628 | egl-9(sa307);kyEx1525[H20::egl-9::SL2gfp] | C. Bargmann (Rockefeller) |
| CX8630 | egl-9(sa307);kyEx1527[myo-3::egl-9::SL2gfp] | C. Bargmann (Rockefeller) |
| CX8756 | egl-9(sa307);kyEx1593[egl-9::egl-9::SL2gfp] | C. Bargmann (Rockefeller) |
| CX9778 | egl-9(sa307);kyEx2159[col-19::egl-9::SL2gfp] | C. Bargmann (Rockefeller) |
| GR366 | eri-1(mg366) | G. Ruvkun (MGH) |
| HY | egl-9(sa307); yeIs[cpr-1::gfp] | R. Aroian (UCSD) |
| HY | egl-9(sa307); yeIs[cpr-1::egl-9] | R. Aroian (UCSD) |
| JIN1381 | vhl-1(ok161); agIs26 [myo-2::mCherry,clec-60::gfp] | This work |
| JIN1574 | egl-9(sa307); agIs26 [myo-2::mCherry,clec-60::gfp] | This work |
| JIN1577 | hif-1(ia4); agIs26 [myo-2::mCherry,clec-60::gfp] | This work |
| JIN1589 | egl-9(sa307); agEx99[myo-2::mCherry,ilys-3::gfp] | This work |
| JT307 | egl-9(sa307) V. | Stock center (CGC) |
| LE436 | swan-1(ok267) V. | Stock center (CGC) |
| MT1216 | egl-9(n586ts) V. | Stock center (CGC) |
| N2 Bristol | wild type | Stock center (CGC) |
| ZG31 | hif-1(ia4) V. | Stock center (CGC) |
| ZG429 | hif-1(ia4); iaIs28[hif-1::myc-hif-1] | J. A. Powell-Coffman (ISU) |
| ZG492 | iaIs7*[nhr-57::gfp]* IV; egl-9(ok478) V. | Stock center (CGC) |
| ZG583 | hif-1(ia4); iaIs34[hif-1::hif-1(P621G)::tag] | J. A. Powell-Coffman (ISU) |
| JT330 | egl-9(sa330) V. | 1. Darby (UCSF) |
| ZG764 | swan-1(ok267) V; iaIs32[hif-1::hif-1(P621G)::tag]; iaIs7[Pnhr-57::GFP] | J. A. Powell-Coffman (ISU) |

**S1b. Bacterial strains.**

| Name | Description |
| --- | --- |
| OP50-1 | non-pathogenic Escherichia coli strain |
| NCTC8325 | S. aureus reference strain |
| HT115 L4440 | RNAi Escherichia coli strain containing empty RNAi plasmid pL4440 |
|  |  |

1. Chang AJ, Bargmann CI (2008) Hypoxia and the HIF-1 transcriptional pathway reorganize a neuronal circuit for oxygen-dependent behavior in Caenorhabditis elegans. Proc Natl Acad Sci USA 105: 7321–7326. doi:10.1073/pnas.0802164105

2. Troemel ER, Chu SW, Reinke V, Lee SS, Ausubel FM, et al. (2006) p38 MAPK regulates expression of immune response genes and contributes to longevity in C. elegans. PLoS Genet 2: e183. doi:10.1371/journal.pgen.0020183

3. Irazoqui JE, Troemel ER, Feinbaum RL, Luhachack LG, Cezairliyan BO, et al. (2010) Distinct pathogenesis and host responses during infection of C. elegans by P. aeruginosa and S. aureus. PLoS Pathog 6: e1000982. doi:10.1371/journal.ppat.1000982
